# Supplementary material for: Development of Novel Class of Phenylpyrazolo[3,4-d]pyrimidine-Based Analogs with Potent Anticancer Activity and Multitarget Enzyme Inhibition Supported by Docking Studies
Source: Int J Mol Sci. 2023 Oct 9;24(19):15026. doi: 10.3390/ijms241915026 (PMC10573254; doi:10.3390/ijms241915026)

Supporting information

Development of novel class of phenylpyrazolo[3,4-*d*]pyrimidine based analogs with potent anticancer activity and multitarget enzyme inhibition supported by docking studies

CONTENTS:

Spectroscopic data of target compounds in MS ……………...……………………..1

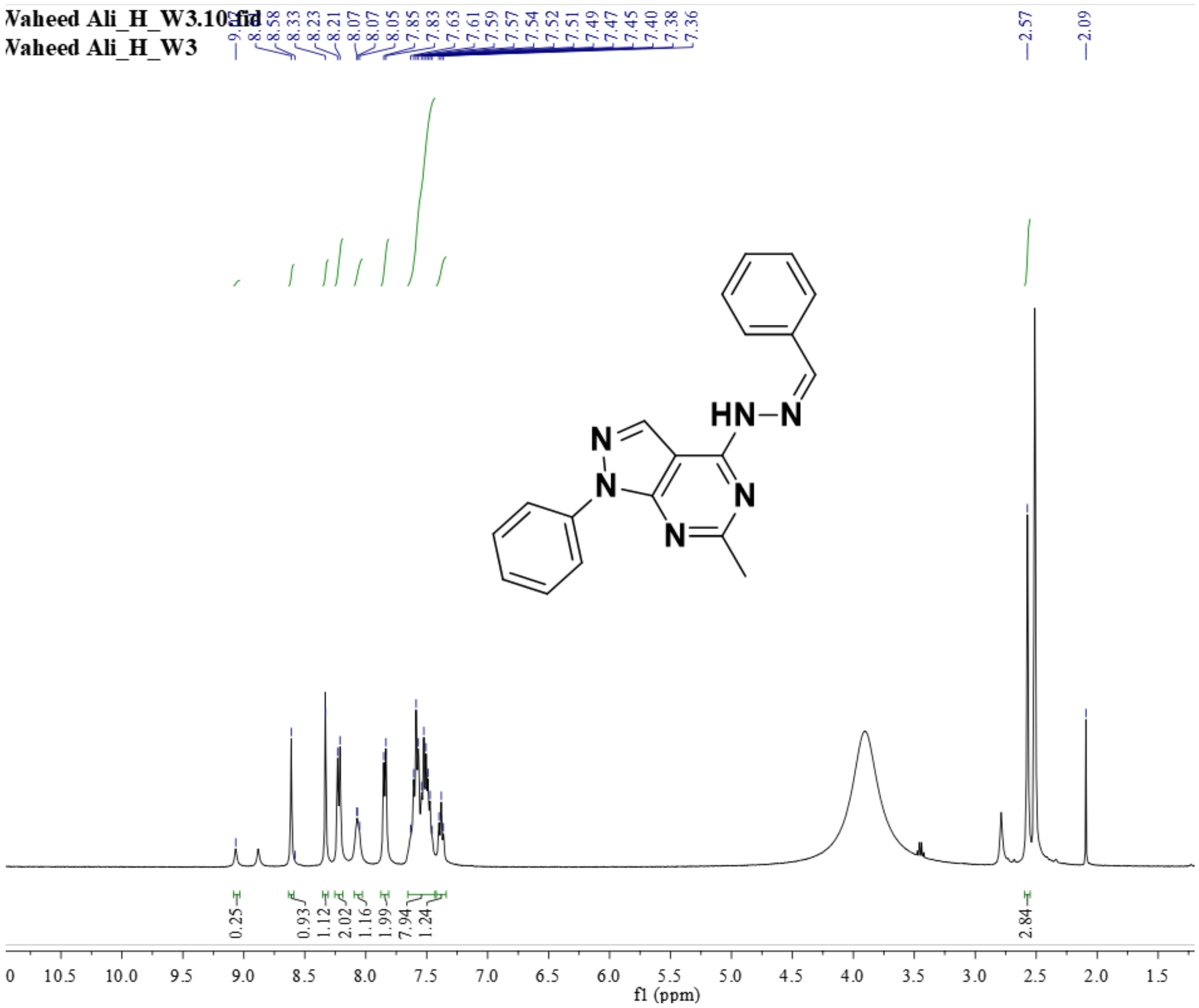

^{1}H NMR of Compound 5a

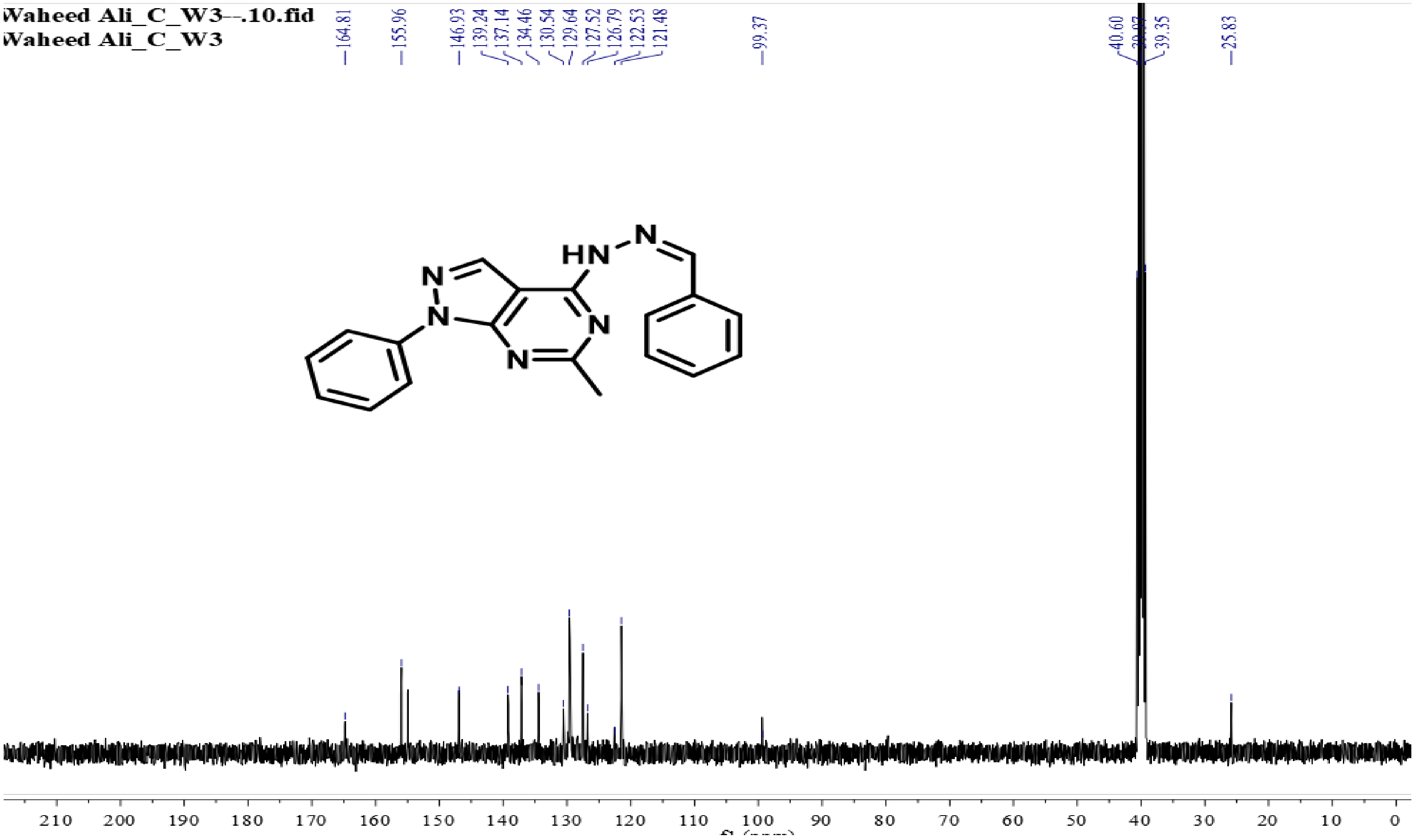

^{13}C NMR of Compound 5a

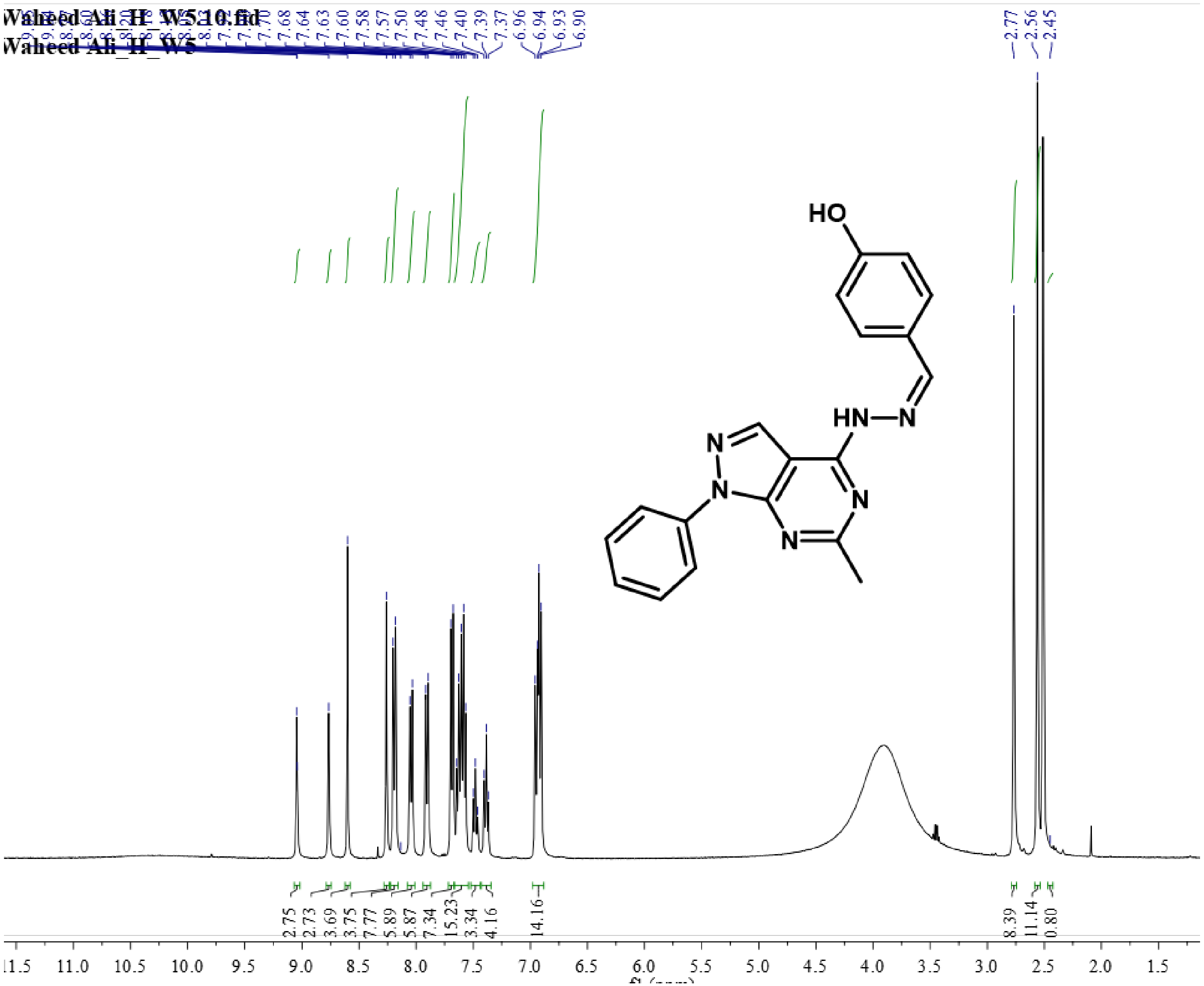

^{1}H NMR of Compound 5b

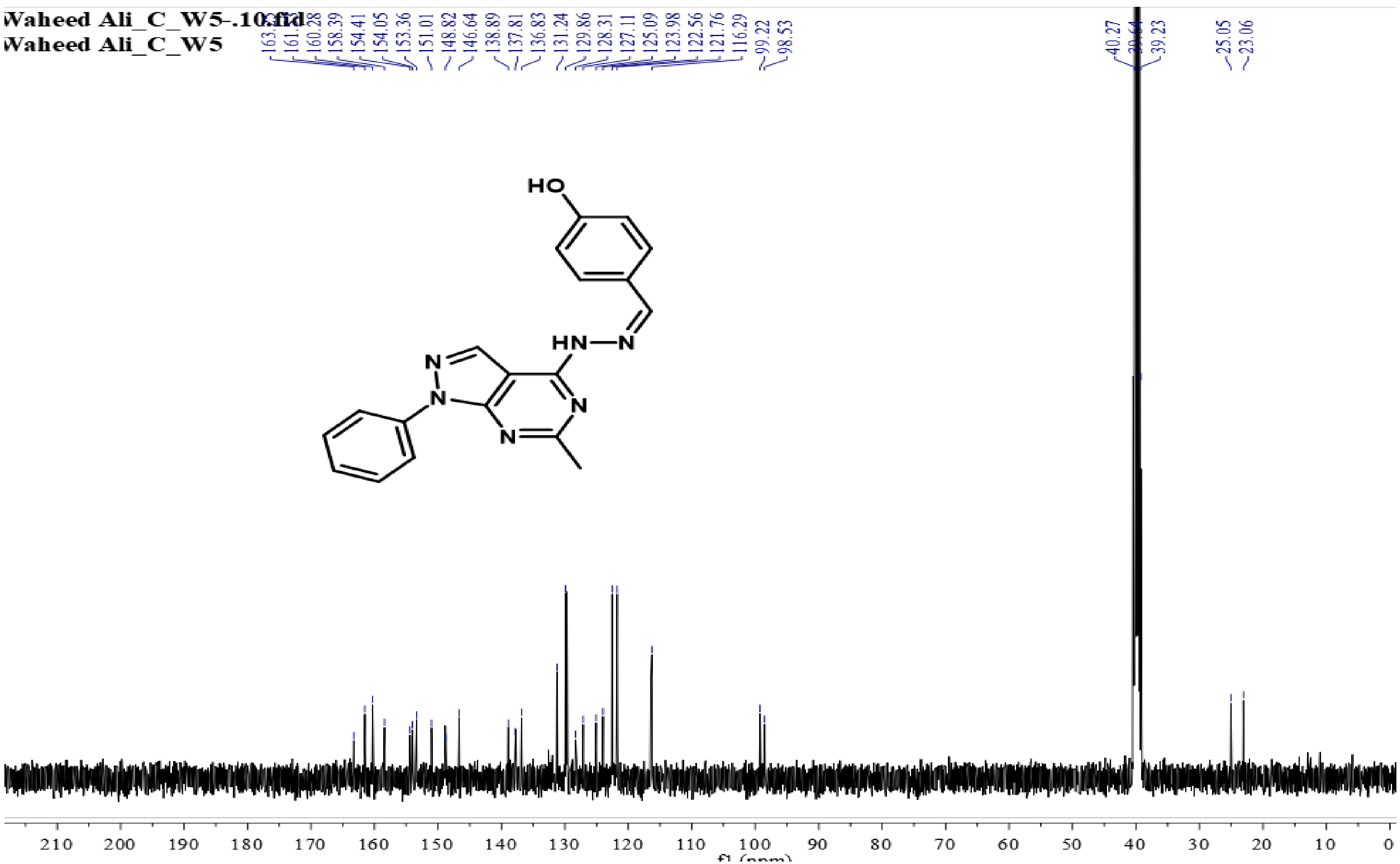

^{13}C NMR of Compound 5b

SAMPLE INFORMATION

Sample Name:	W-5	Acquired By:	System
Sample Type:	Unknown	Sample Set Name:	errere
Vial:	21	Acq. Method Set:	FTANinstrument
Injection #:	1	Processing Method:	Default
Injection Volume:	10.00 ul	Channel Name:	215.0nm
Run Time:	26.0 Minutes	Proc. Chnl. Descr.:	996 PDA 215.0 nm (PDA 210.0 to 800.0 nm at
Date Acquired:	9/24/2023 2:01:03 AM EEST		
Date Processed:	9/24/2023 10:20:39 AM EEST		

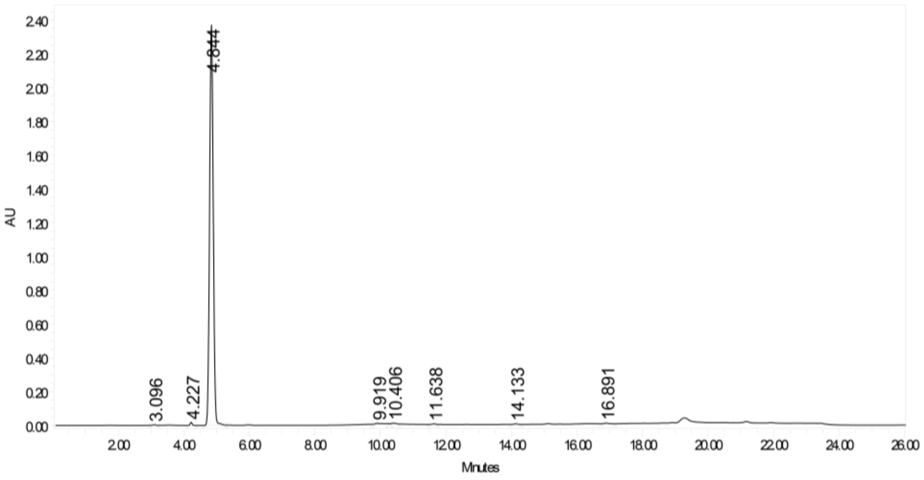

	RT	Area	%Area	Height
1	3.096	13236	0.07	2894
2	4.227	77241	0.42	18030
3	4.844	18186264	98.23	2369496
4	9.919	93614	0.51	5337
5	10.406	56579	0.31	5740
6	11.638	27840	0.15	4057
7	14.133	25287	0.14	3333
8	16.891	33035	0.18	3899

HPLC of Compound 5b

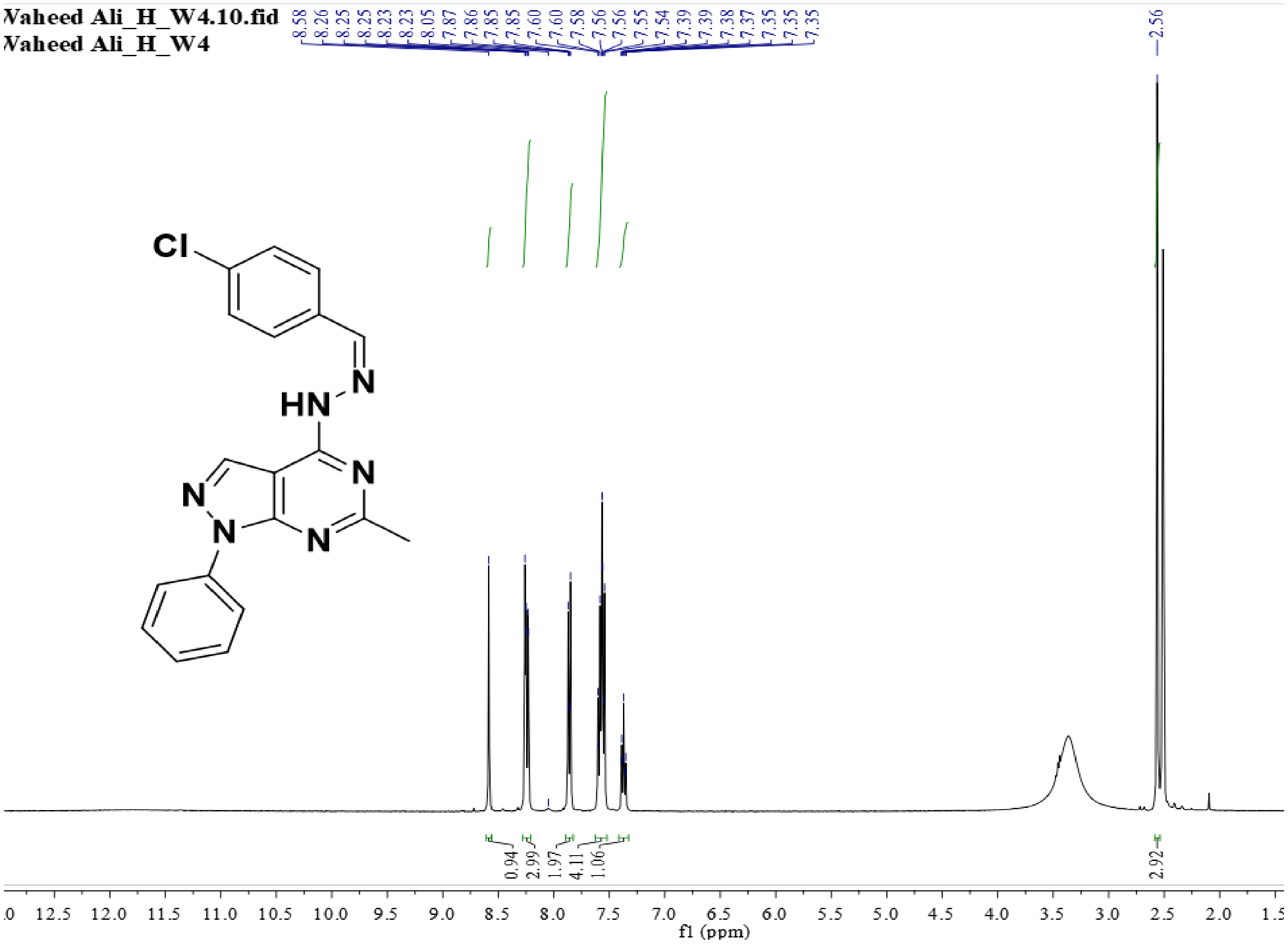

^{1}H NMR of Compound 5c

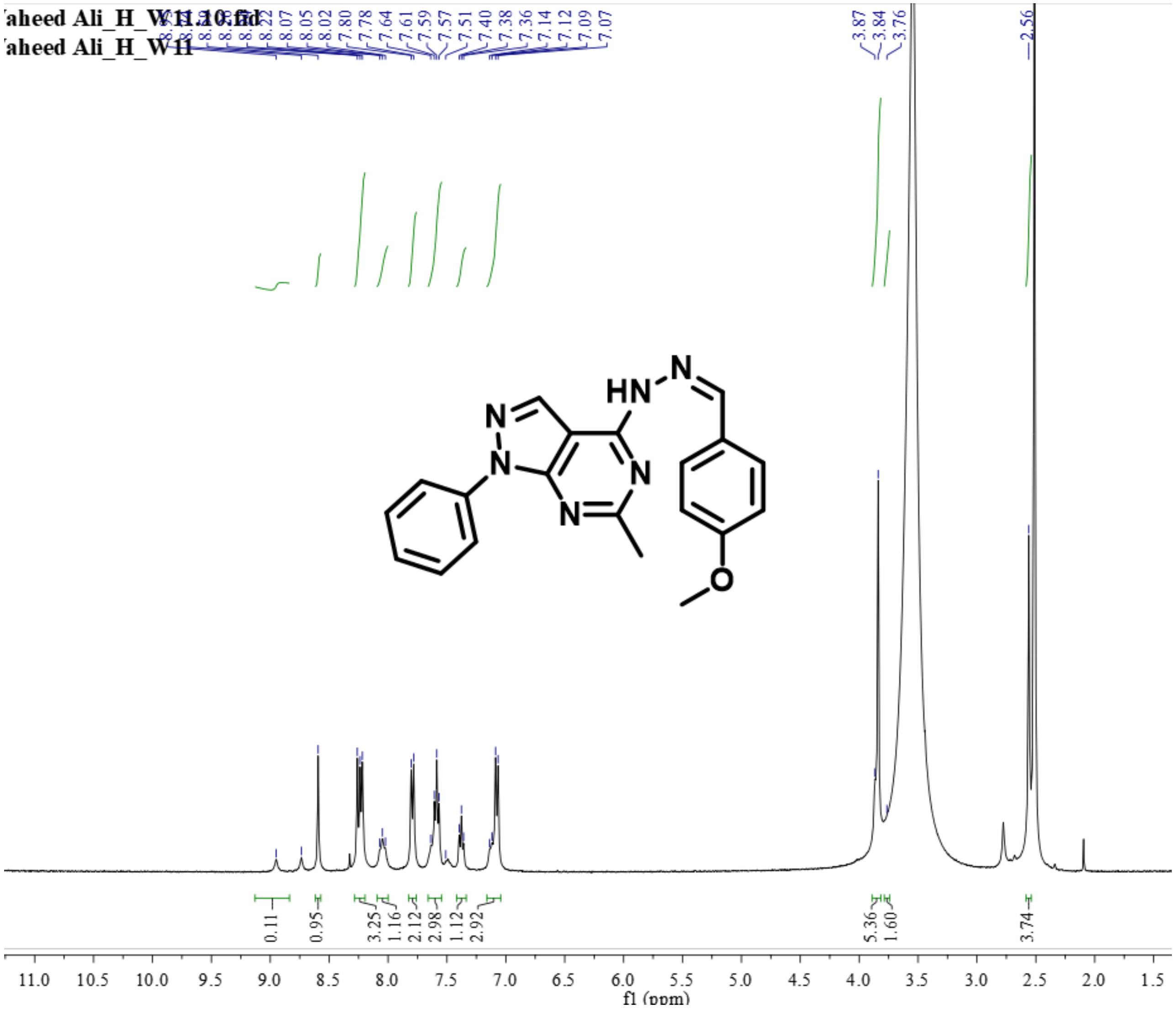

^{1}H NMR of Compound 5d

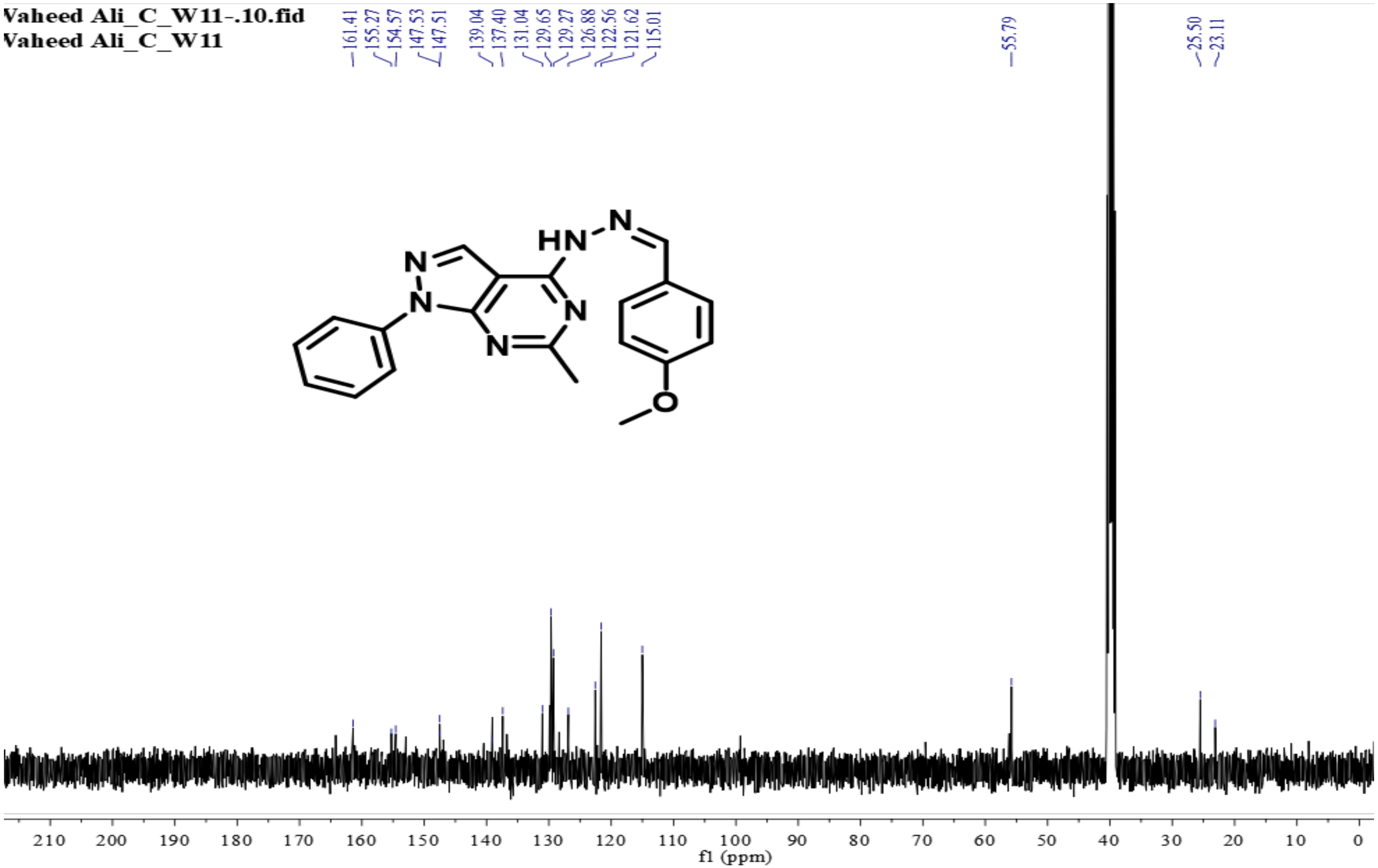

^{13}C NMR of Compound 5d

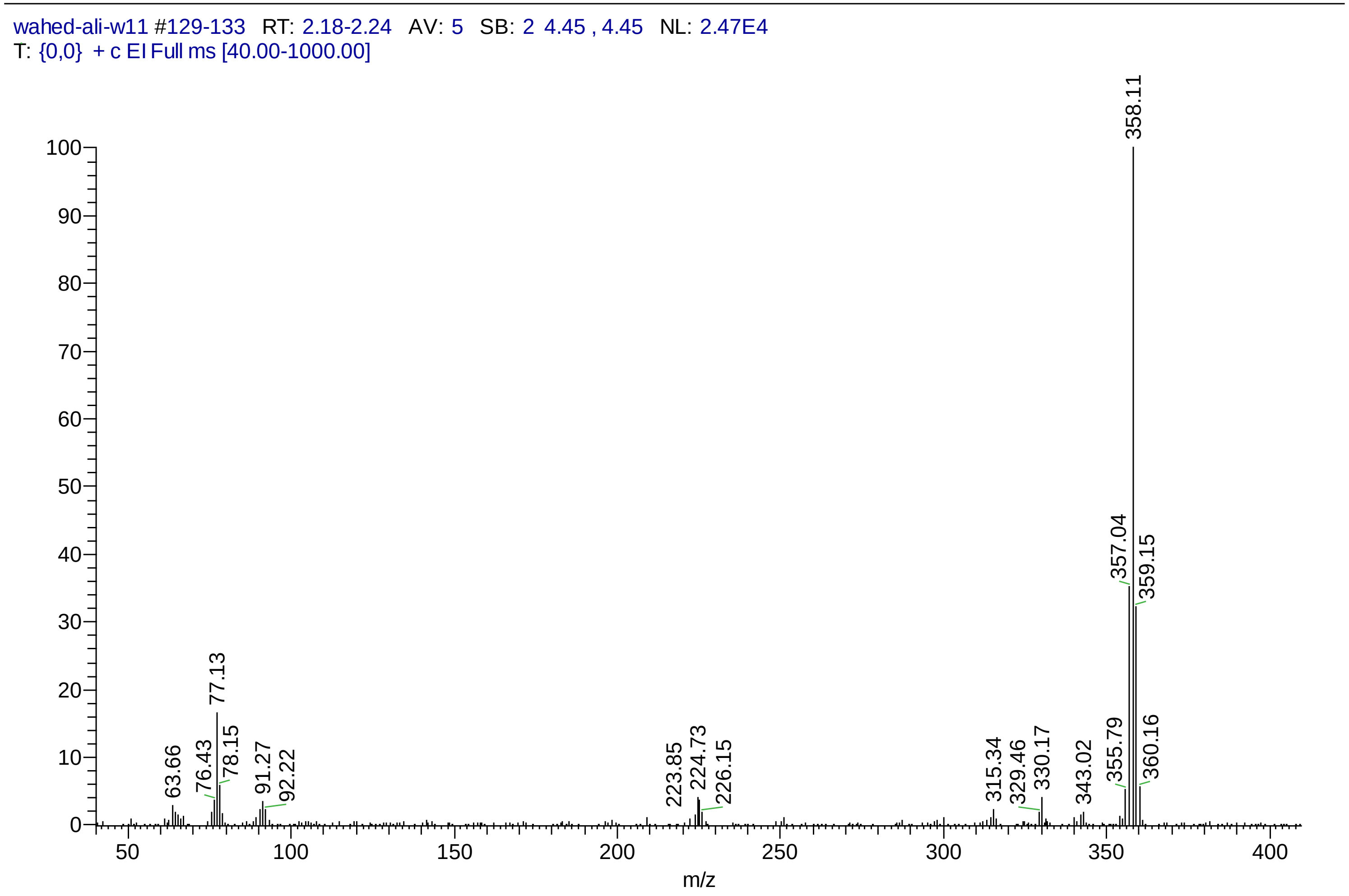

Mass of Compound 5d

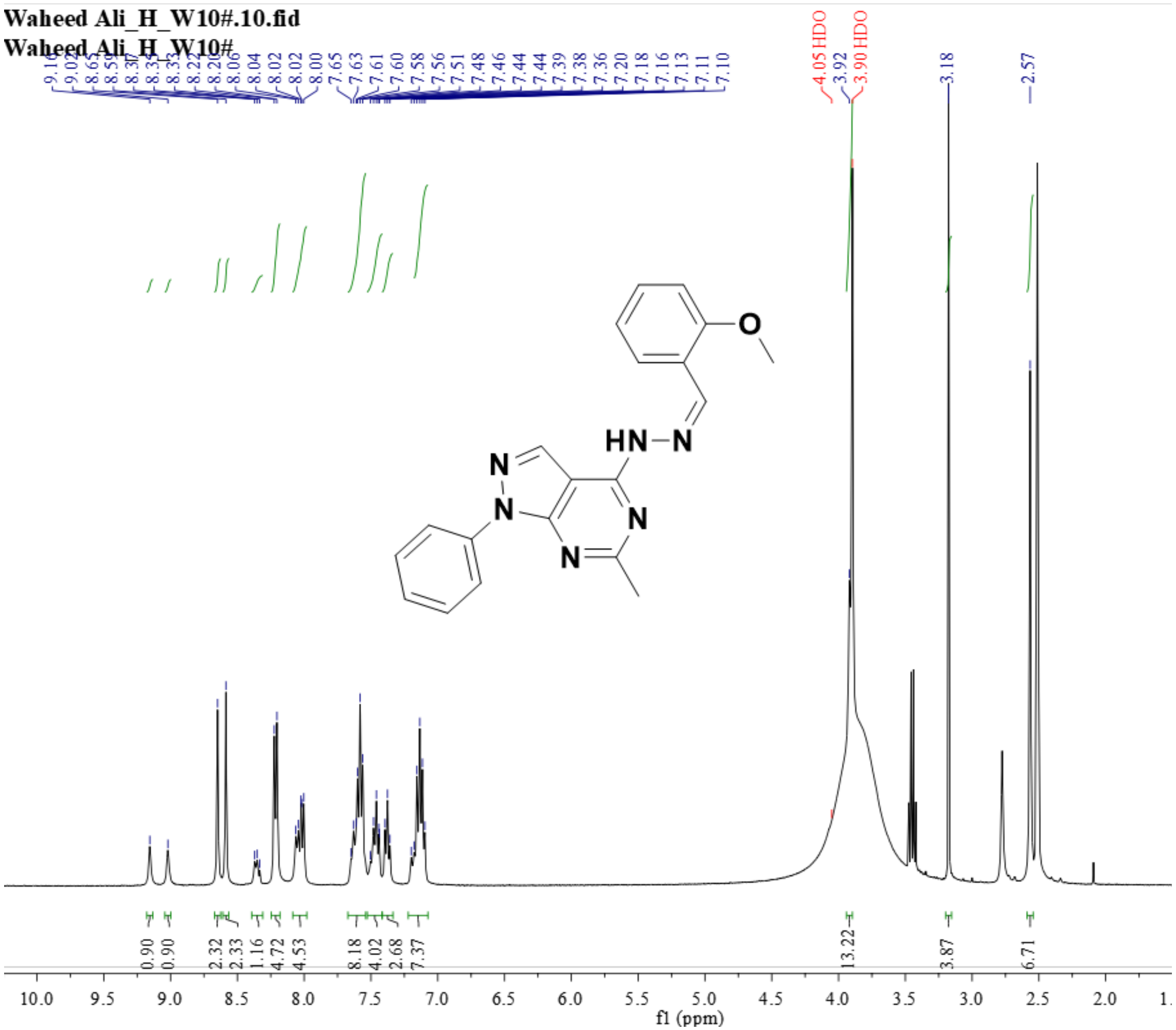

^{1}H NMR of Compound 5e

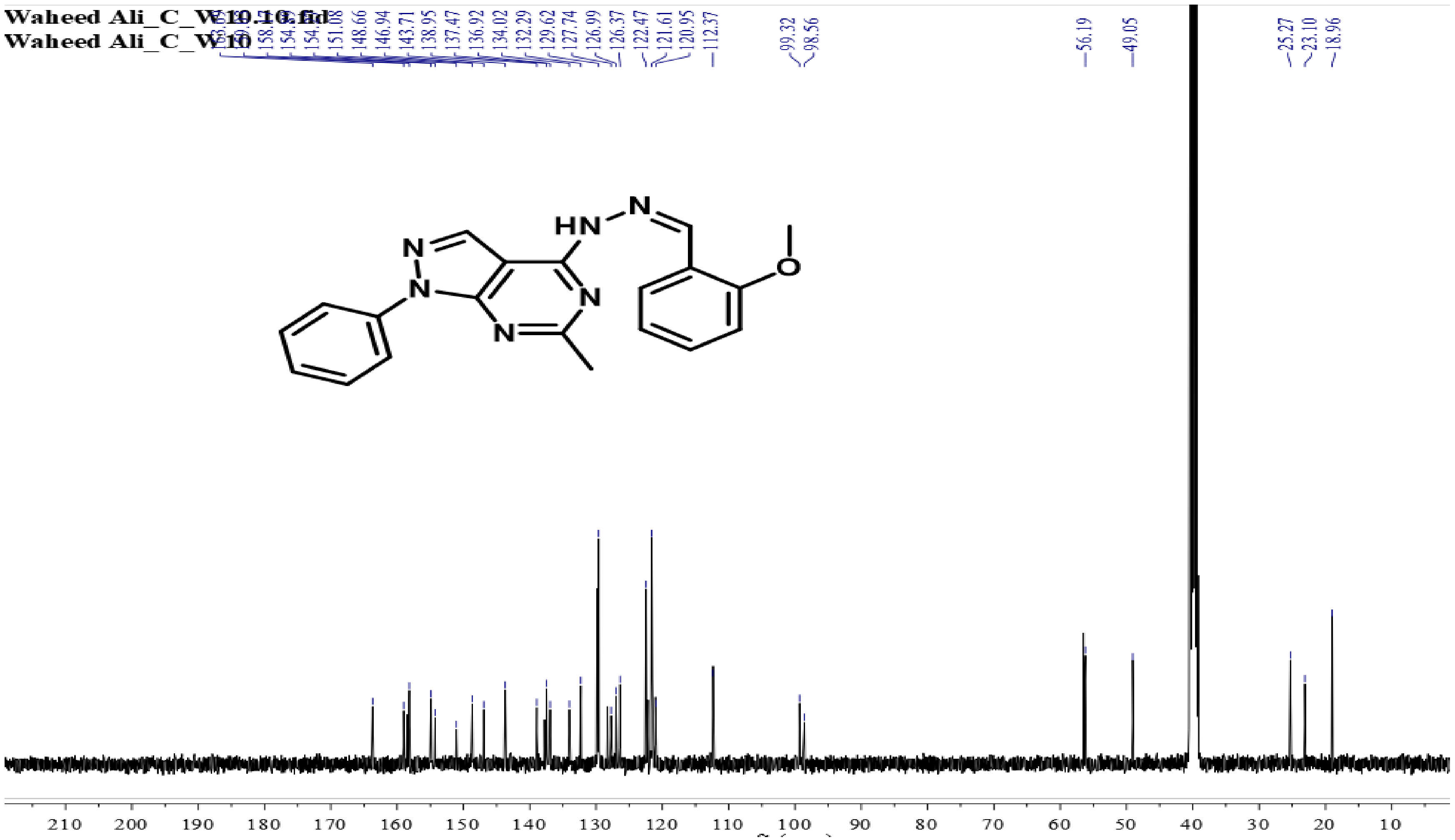

^{13}C NMR of Compound 5e

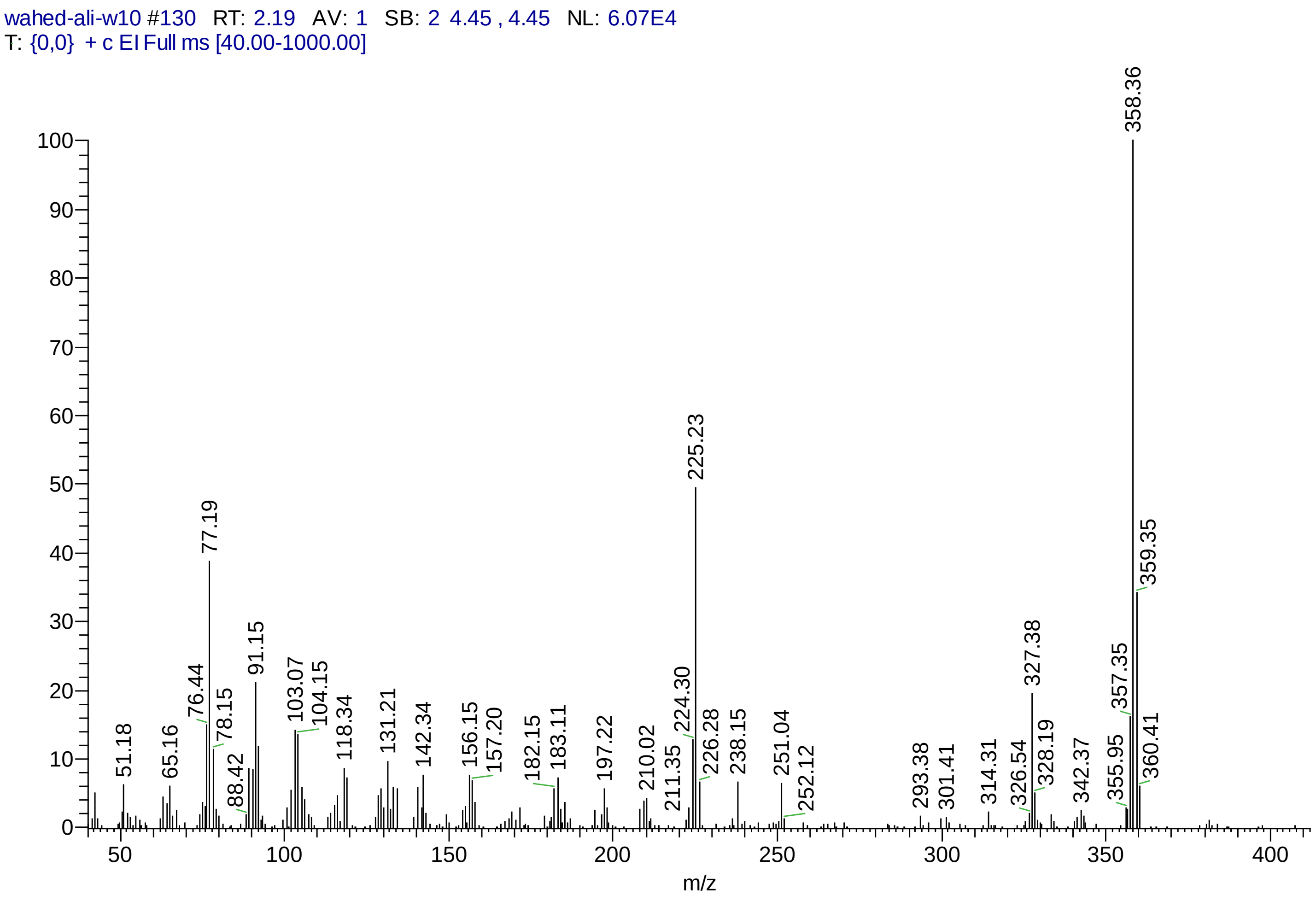

Mass of Compound 5e

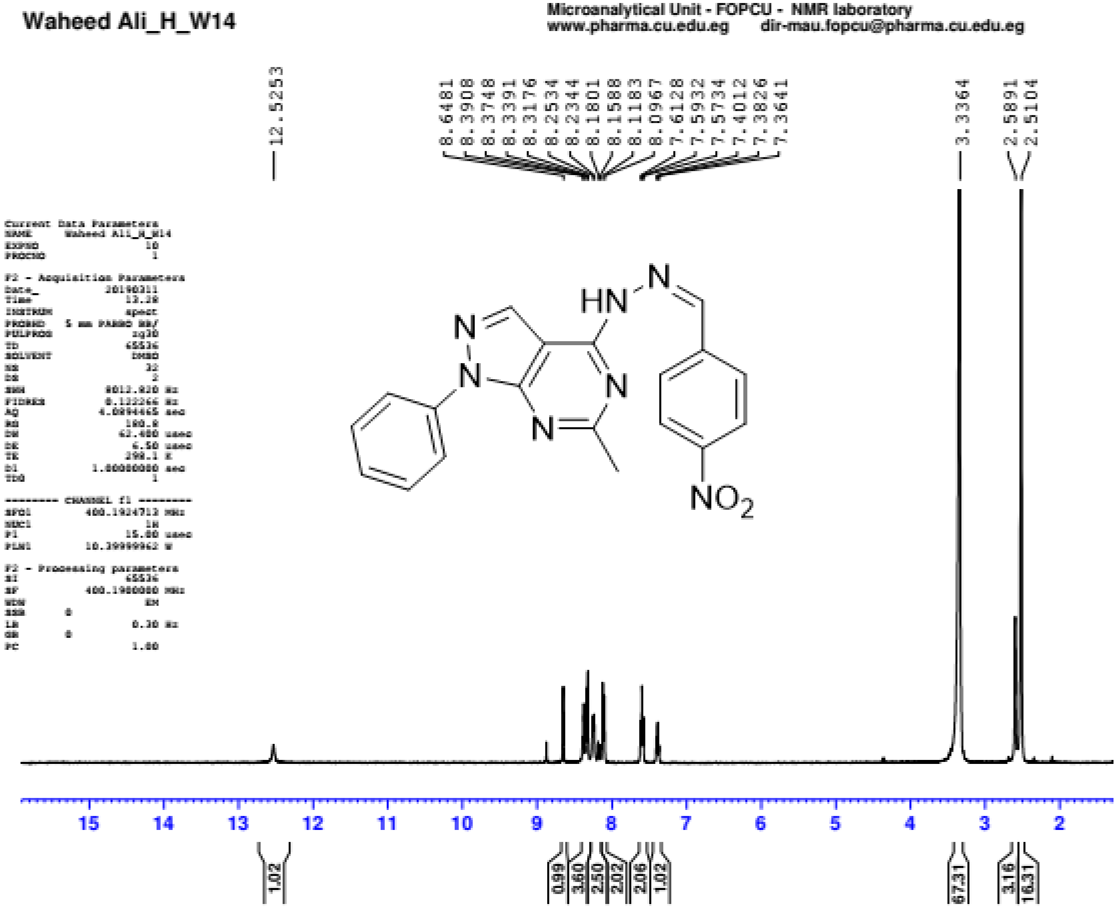

^{1}H NMR of Compound 5f

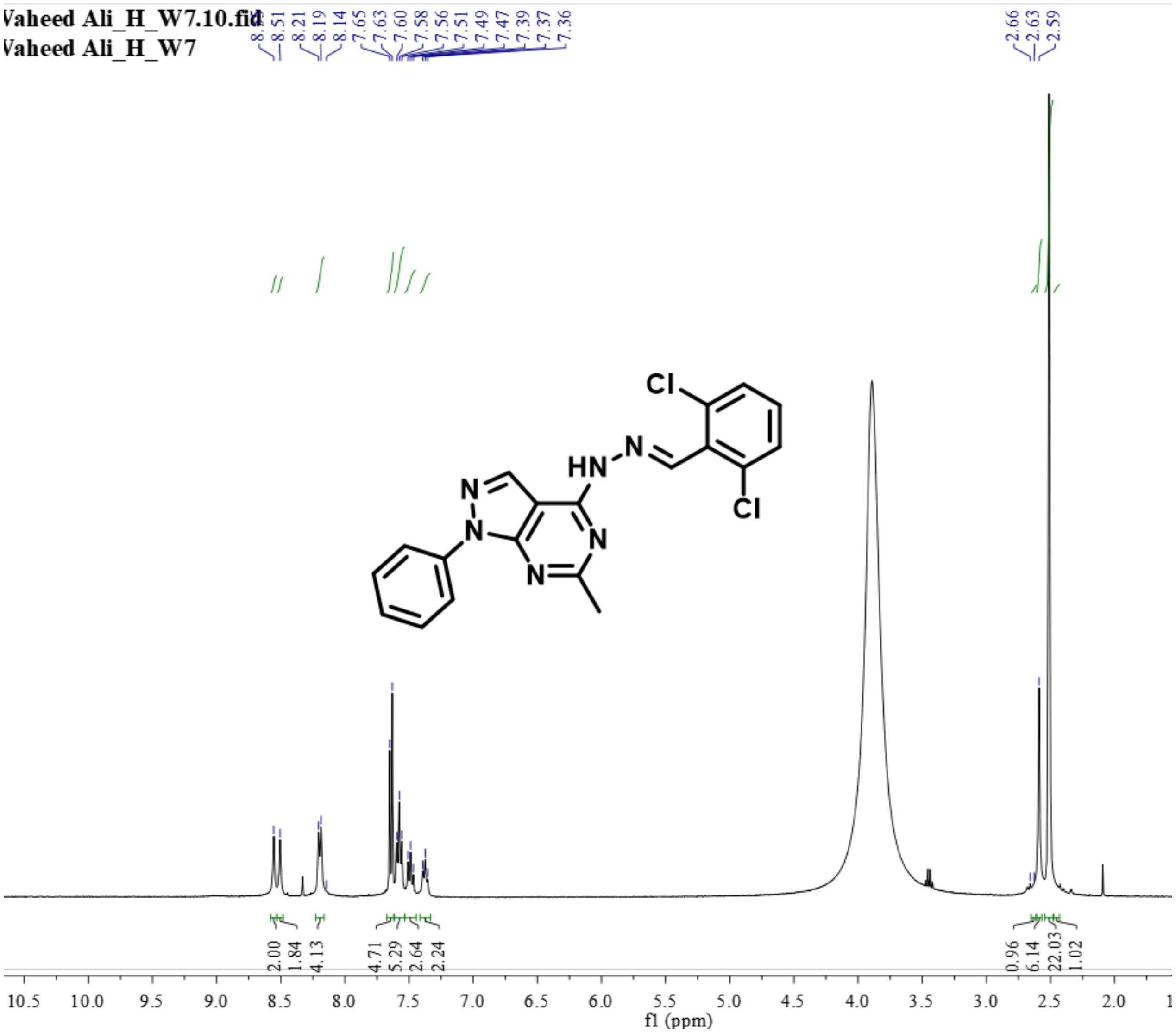

^{1}H NMR of Compound 5g

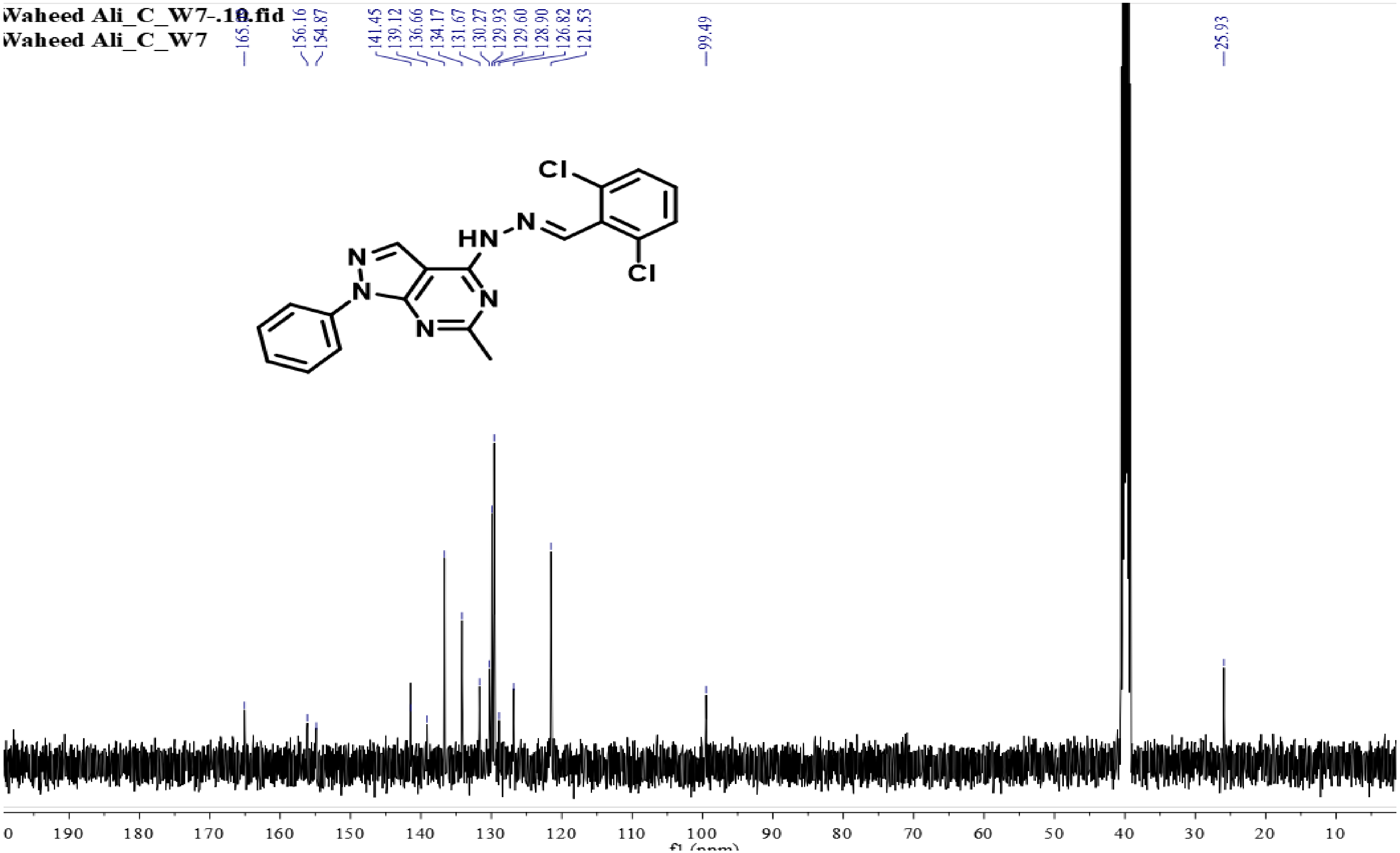

^{13}C NMR of Compound 5g

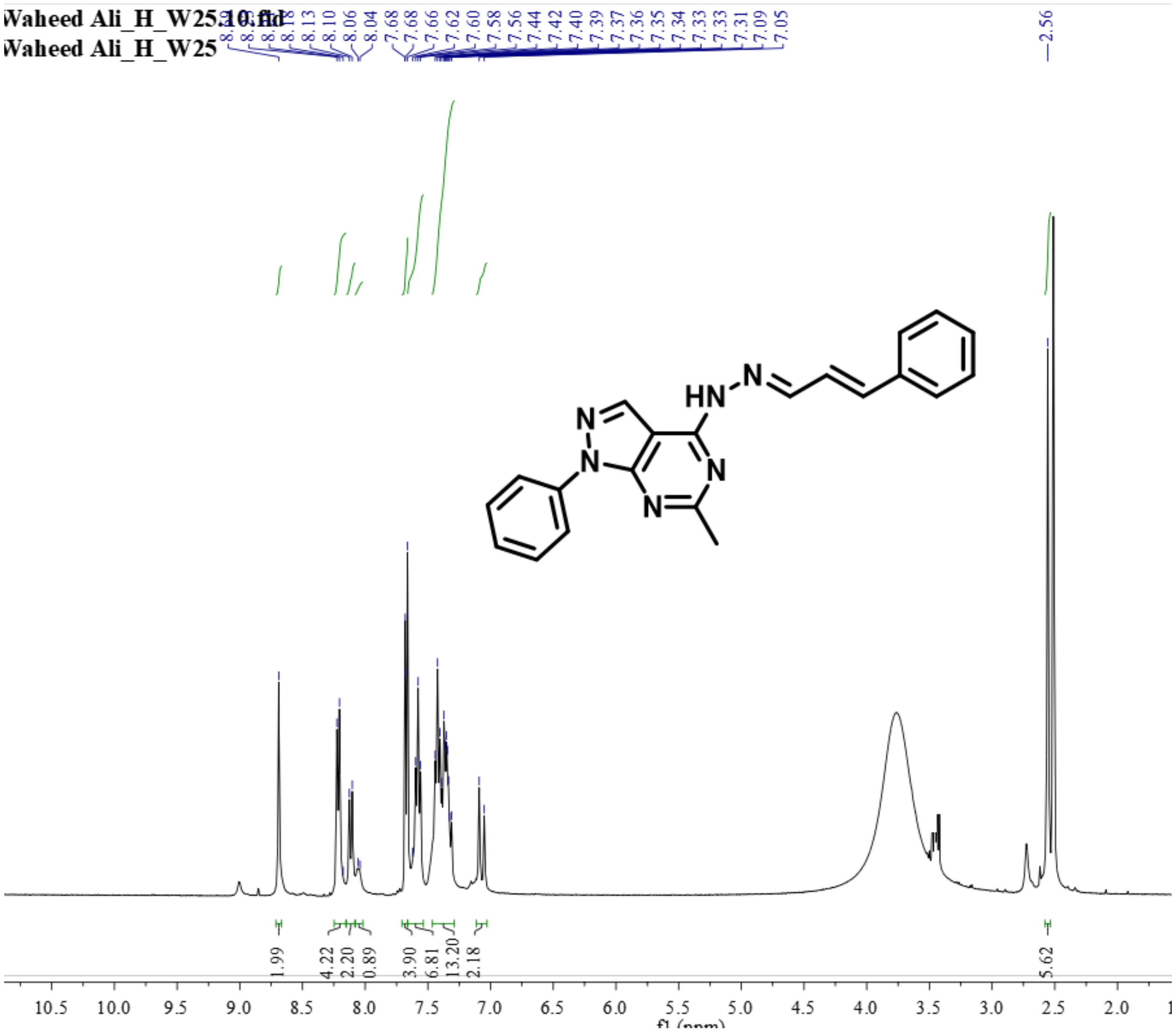

^{1}H NMR of Compound 5h

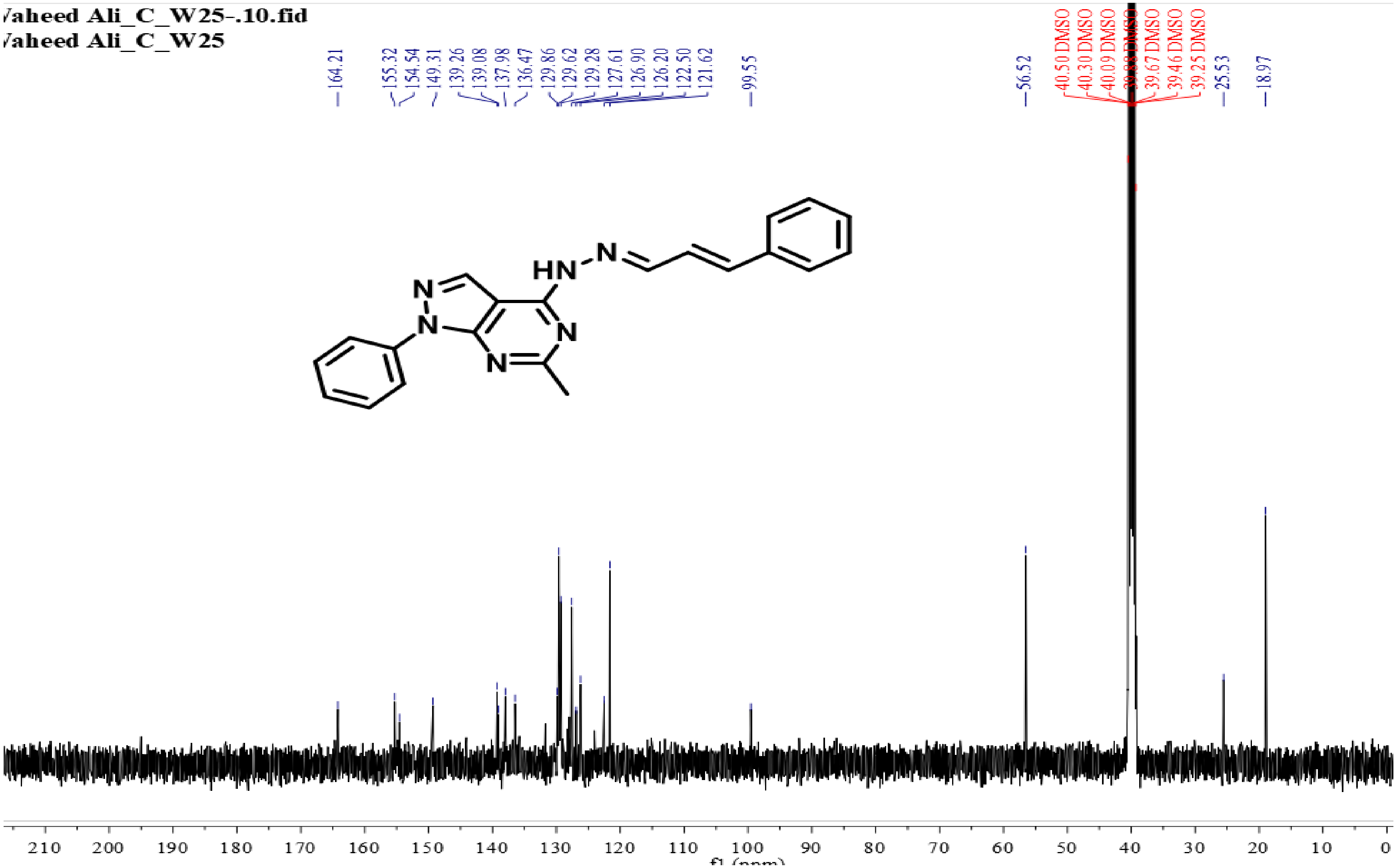

^{13}C NMR of Compound 5h

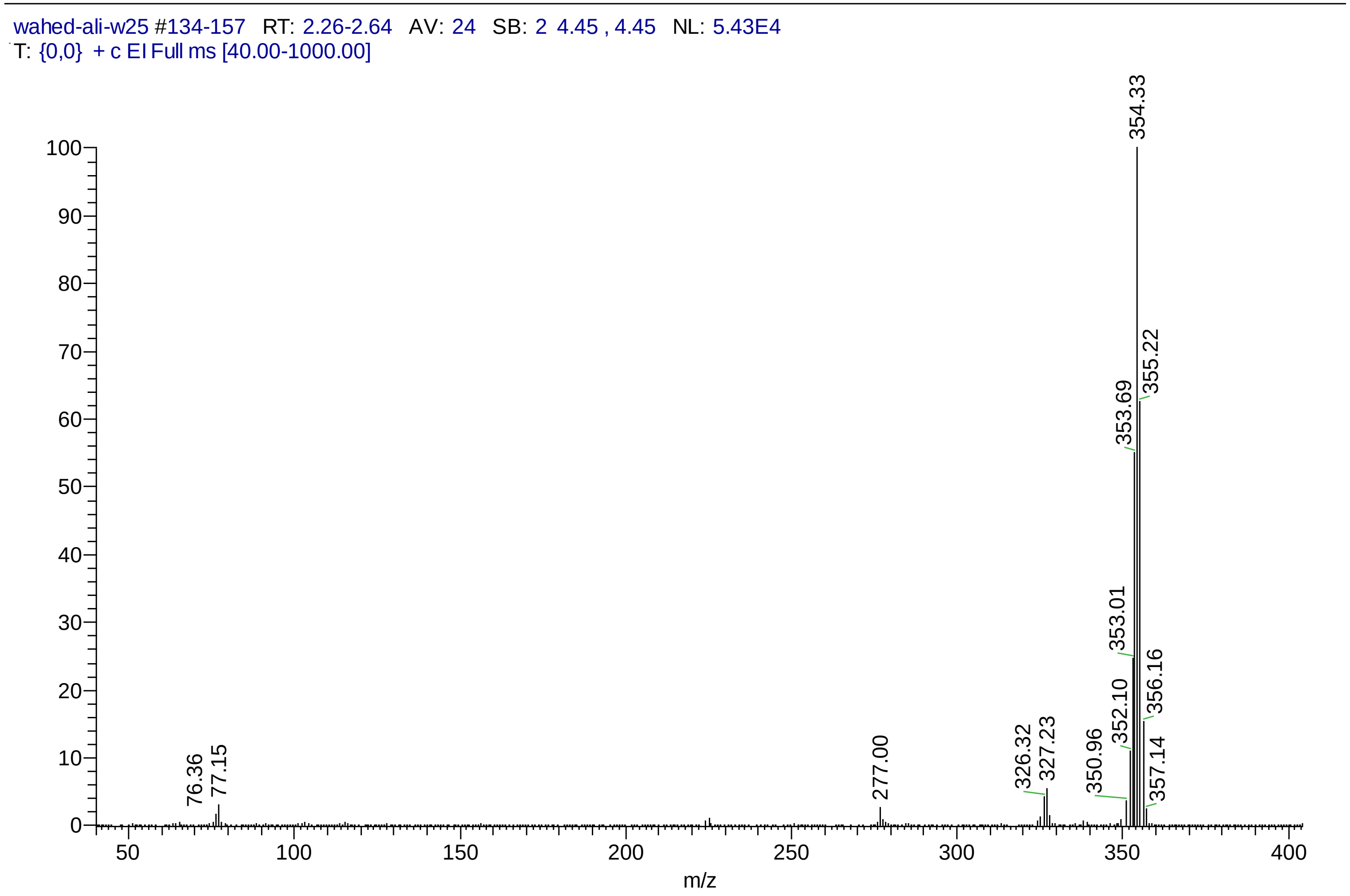

Mass of Compound 5h

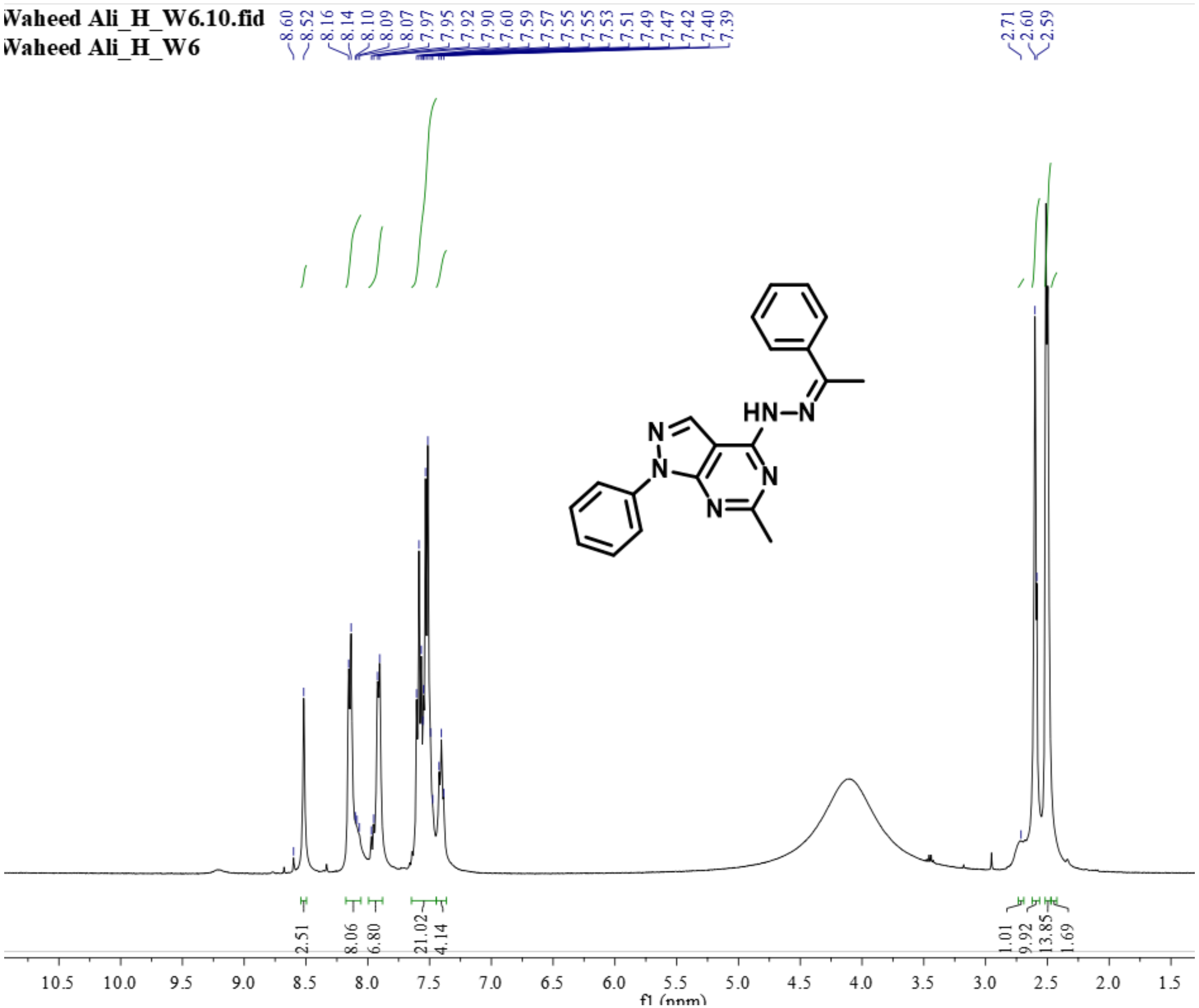

^{1}H NMR of Compound 5i

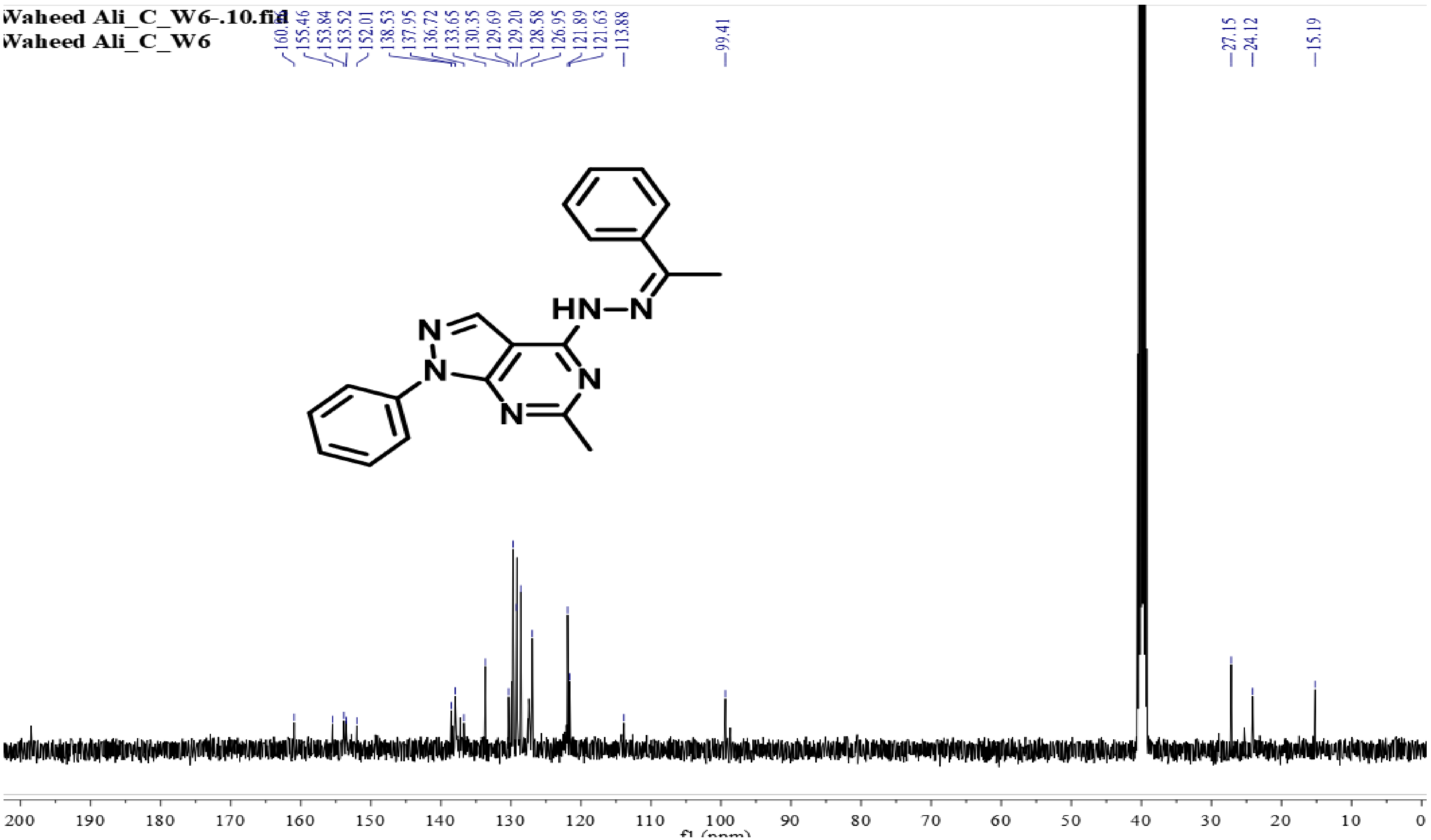

^{13}C NMR of Compound 5i

SAMPLE INFORMATION

Sample Name:	W-6	Acquired By:	System
Sample Type:	Unknown	Sample Set Name:	emere
Vial:	18	Acq. Method Set:	FTANinstrument
Injection #:	1	Processing Method:	Default
Injection Volume:	10.00 ul	Channel Name:	234.0nm
Run Time:	26.0 Minutes	Proc. Chnl. Descr.:	996 PDA 234.0 nm (PDA 210.0 to 800.0 nm at
Date Acquired:	9/24 2023 12:40:00 AM EEST		
Date Processed:	9/24/2023 10:22:58 AM EEST		

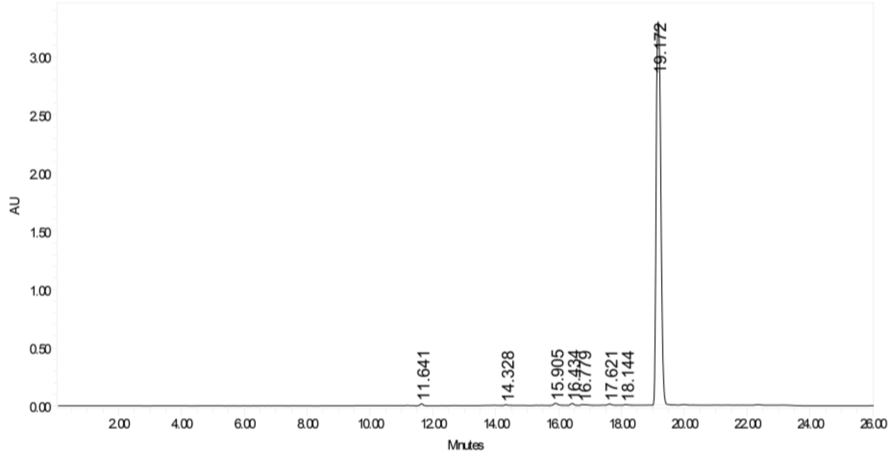

	RT	Area	%Area	Height
1	11.641	111086	0.32	16902
2	14.328	34834	0.10	5515
3	15.905	147448	0.43	17126
4	16.434	116555	0.34	15682
5	16.779	92146	0.27	6877
6	17.621	73783	0.22	9594
7	18.144	40526	0.12	5381
8	19.172	33599443	98.20	3300551

HPLC of Compound 5i

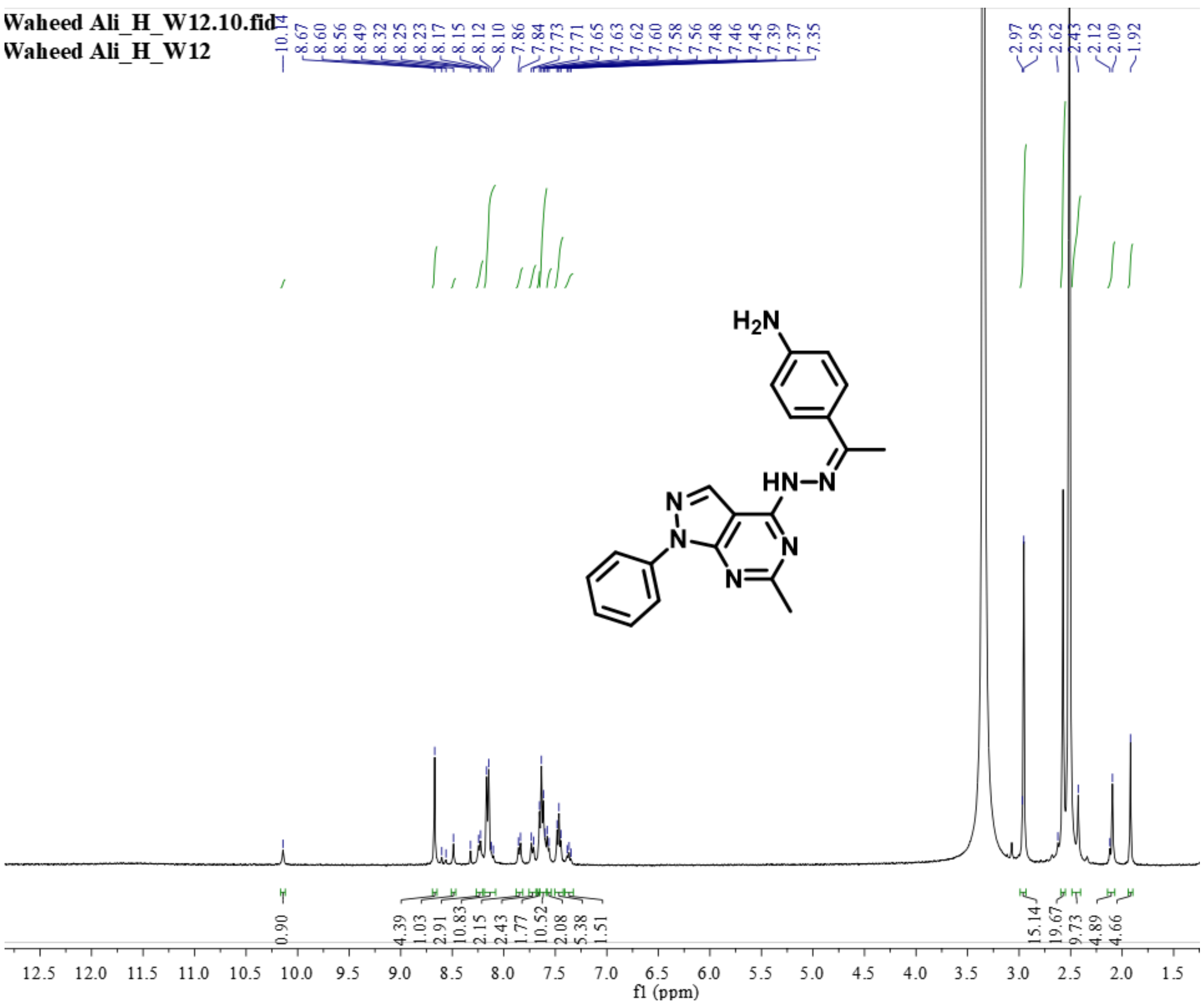

^{1}H NMR of Compound 5j

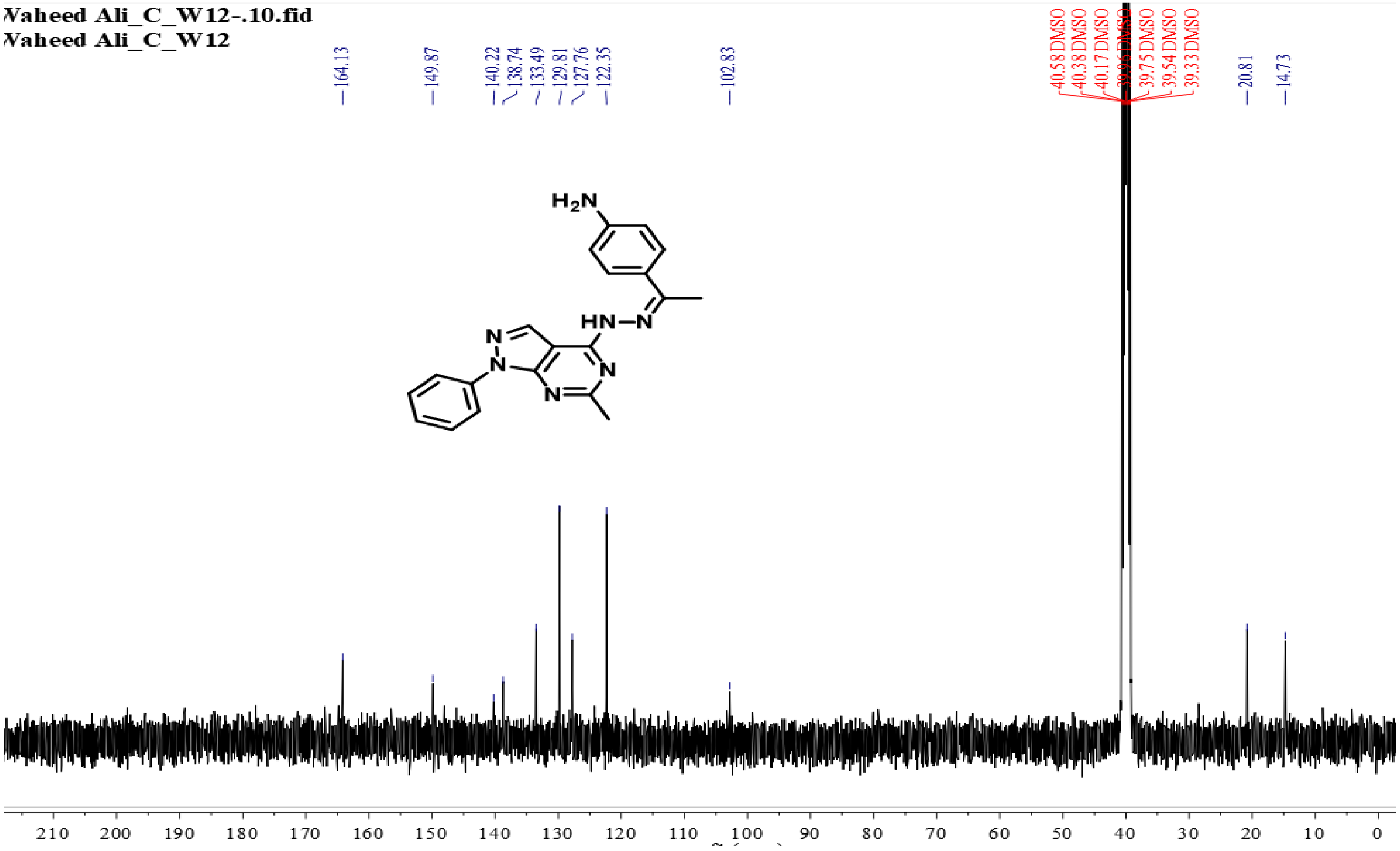

^{13}C NMR of Compound 5j

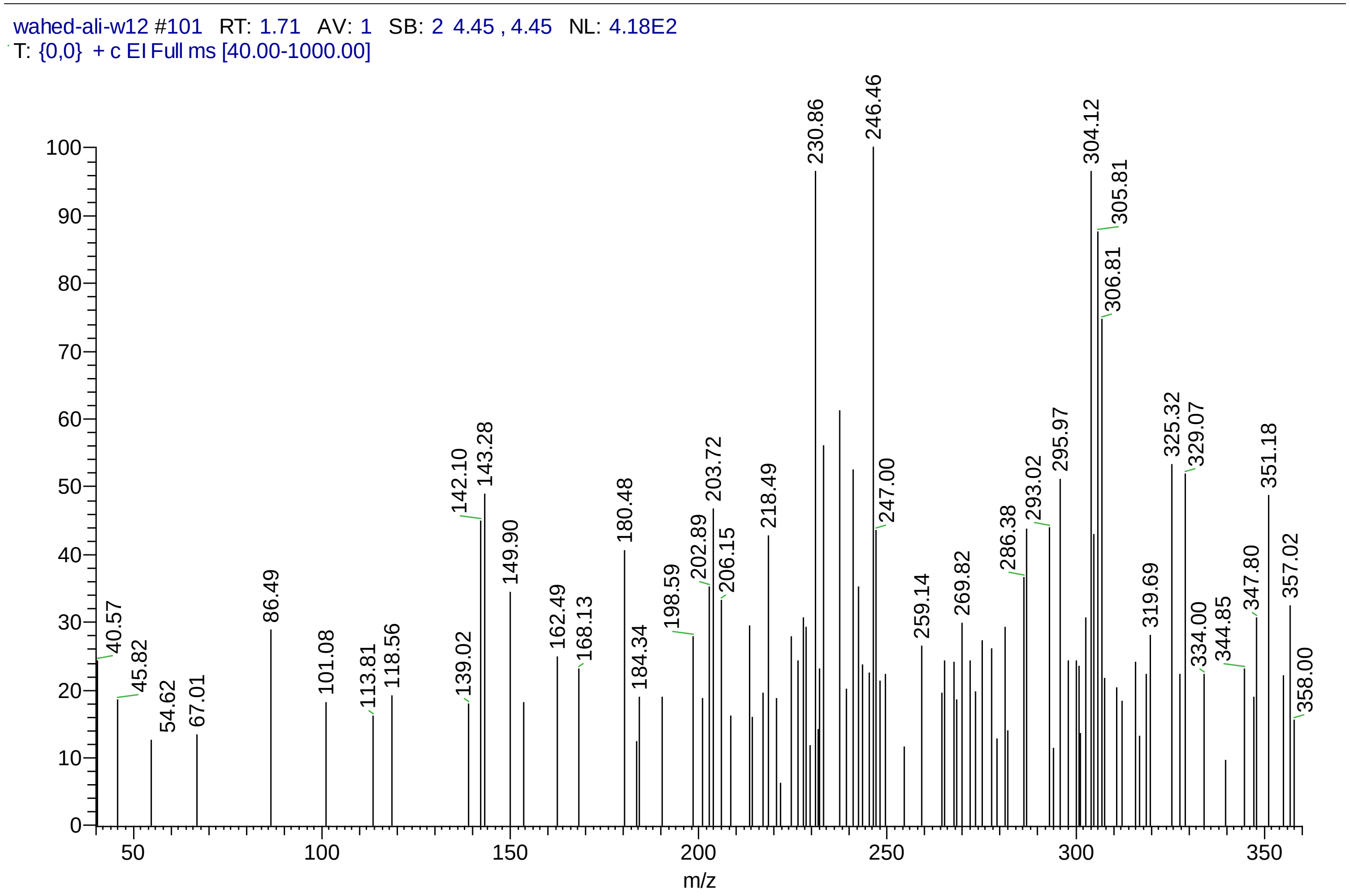

Mass of Compound 5j

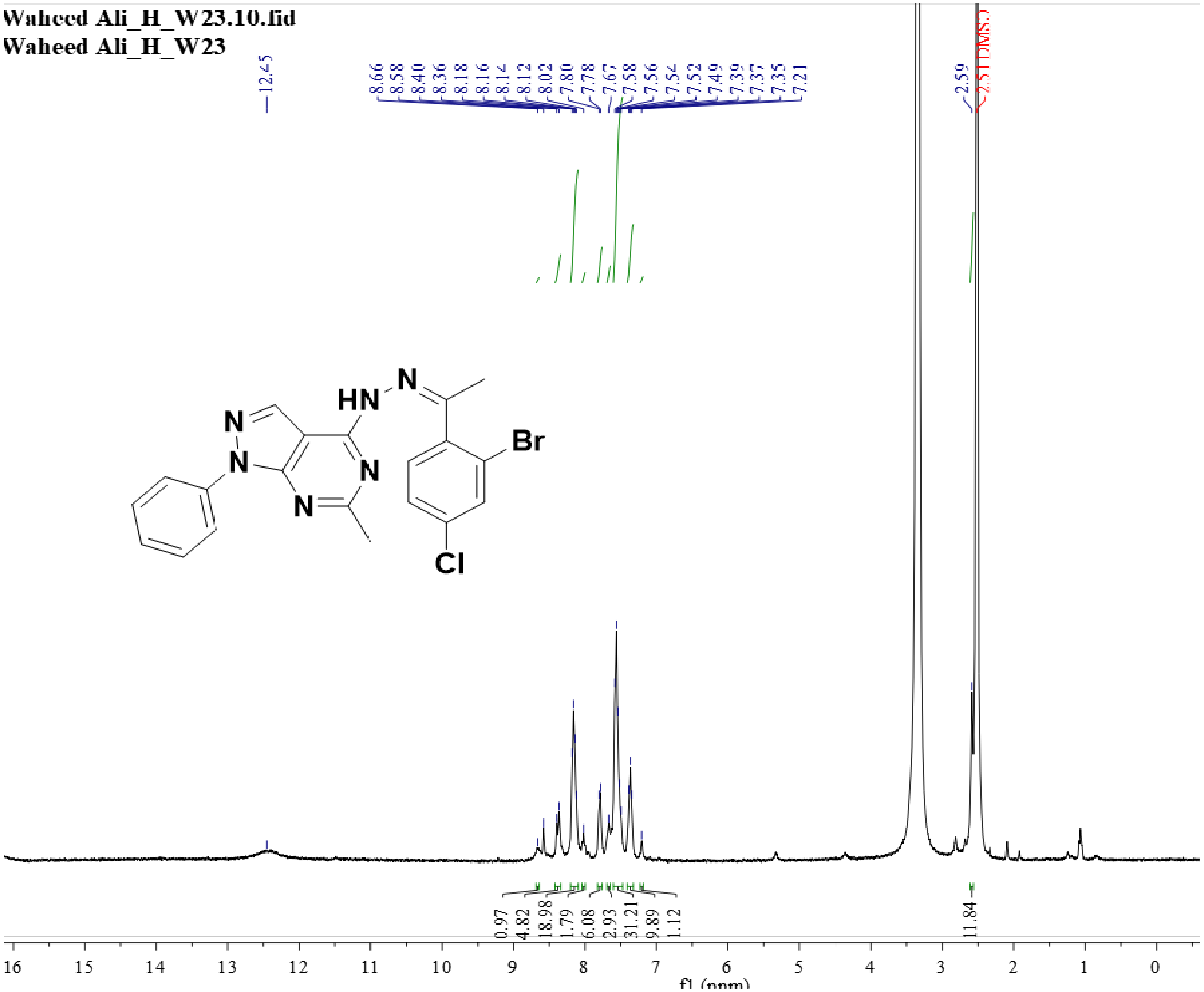

^{1}H NMR of Compound 5l

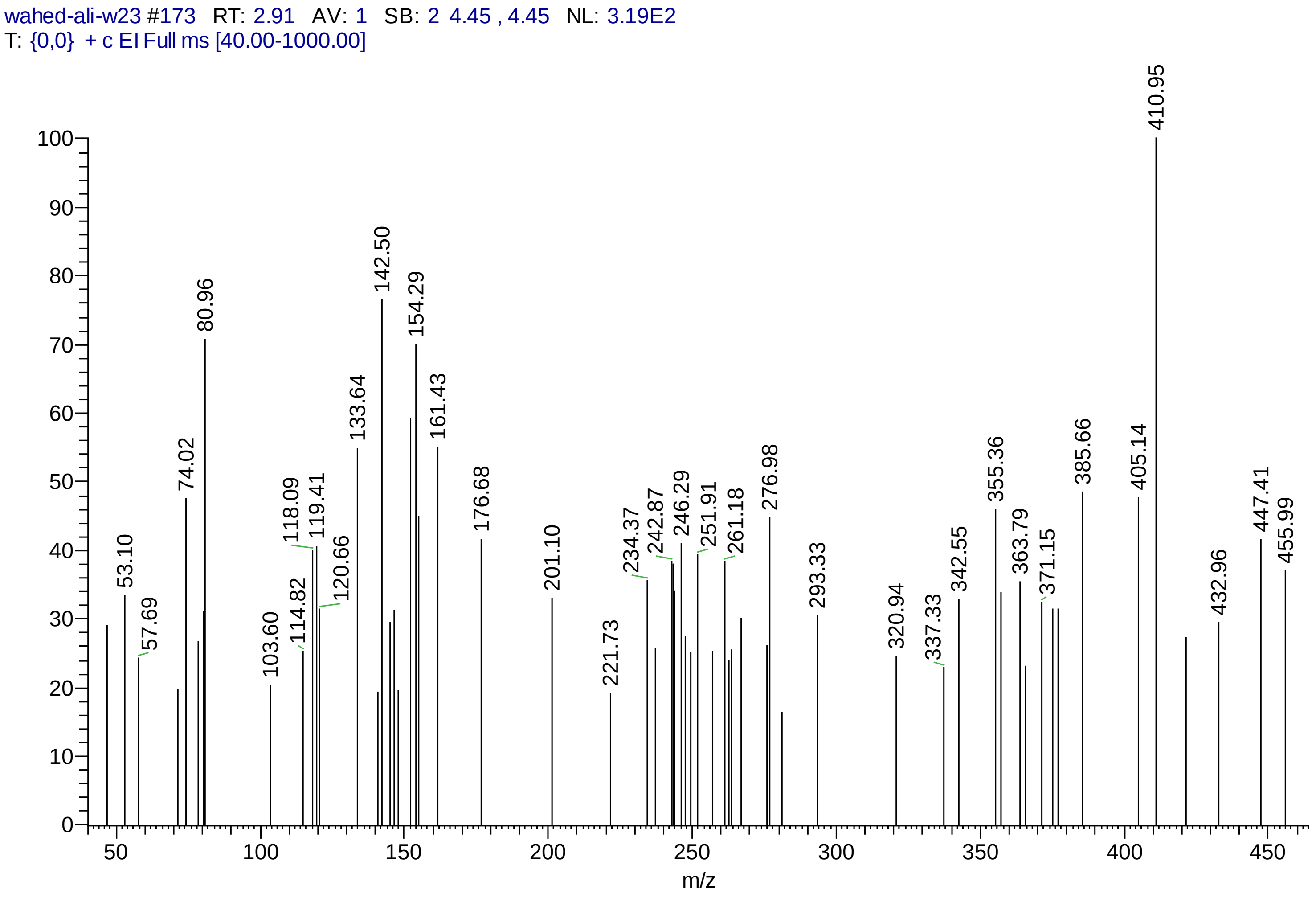

Mass of Compound 5l

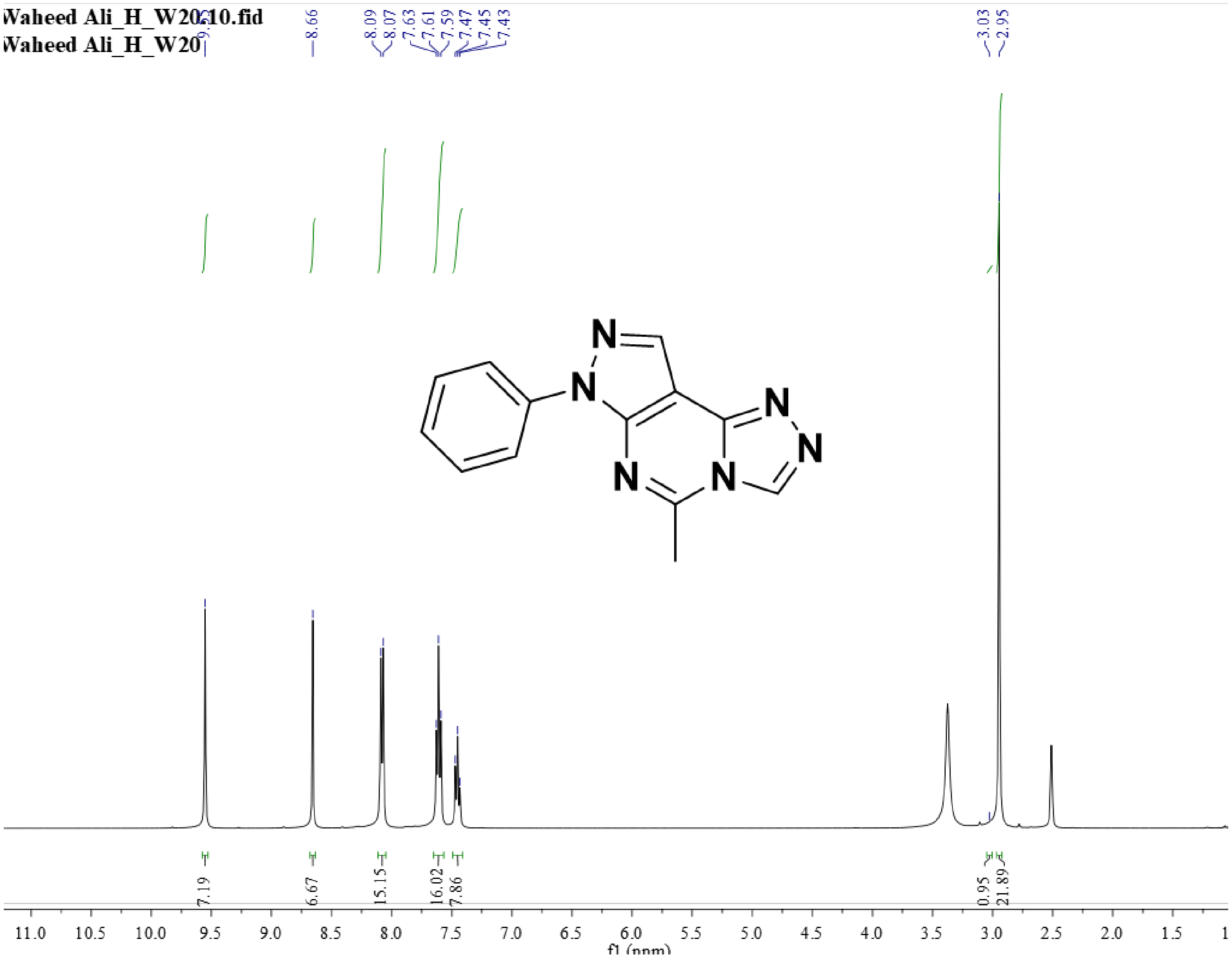

^{1}H NMR of compound 6a

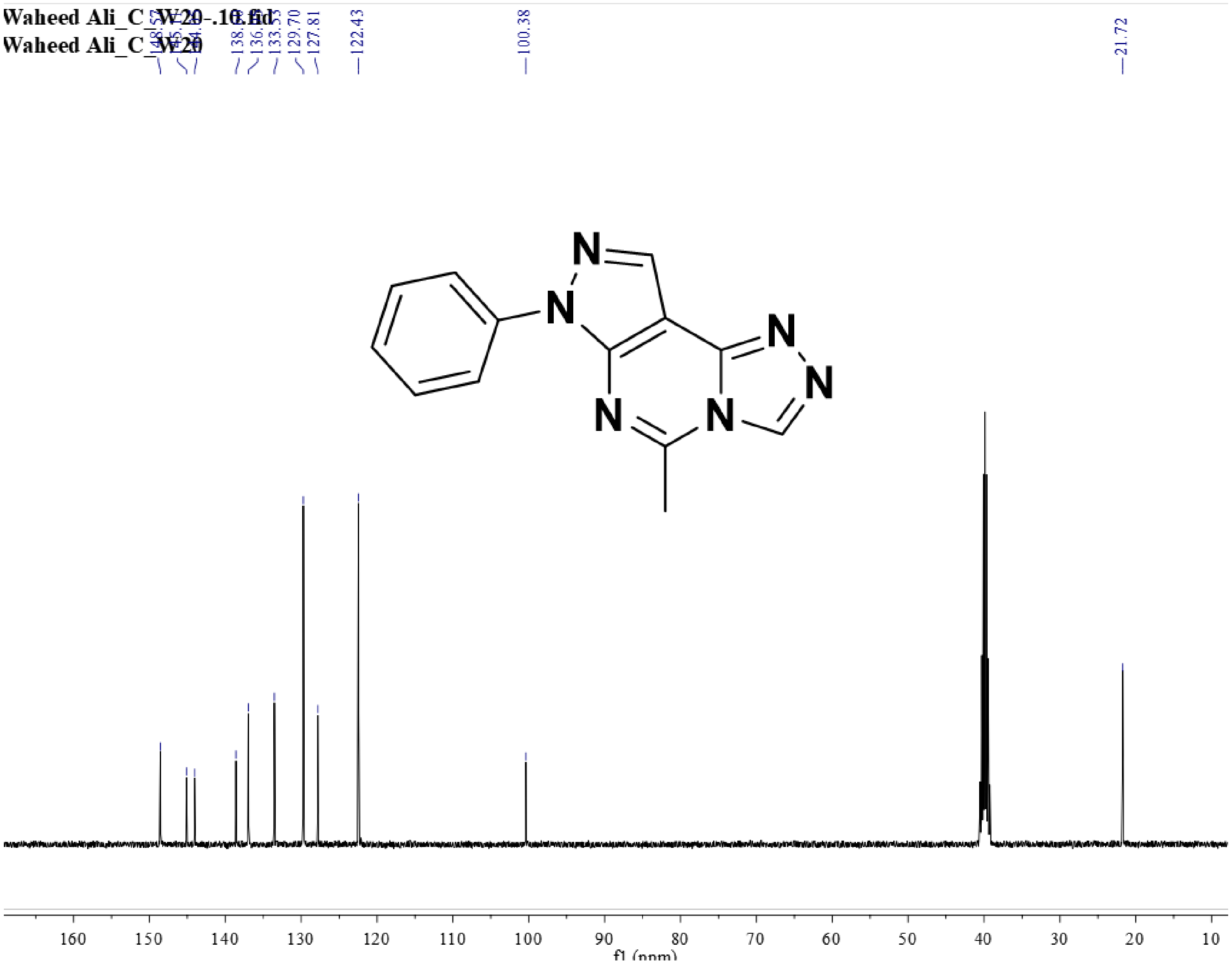

^{13}C NMR of compound 6a

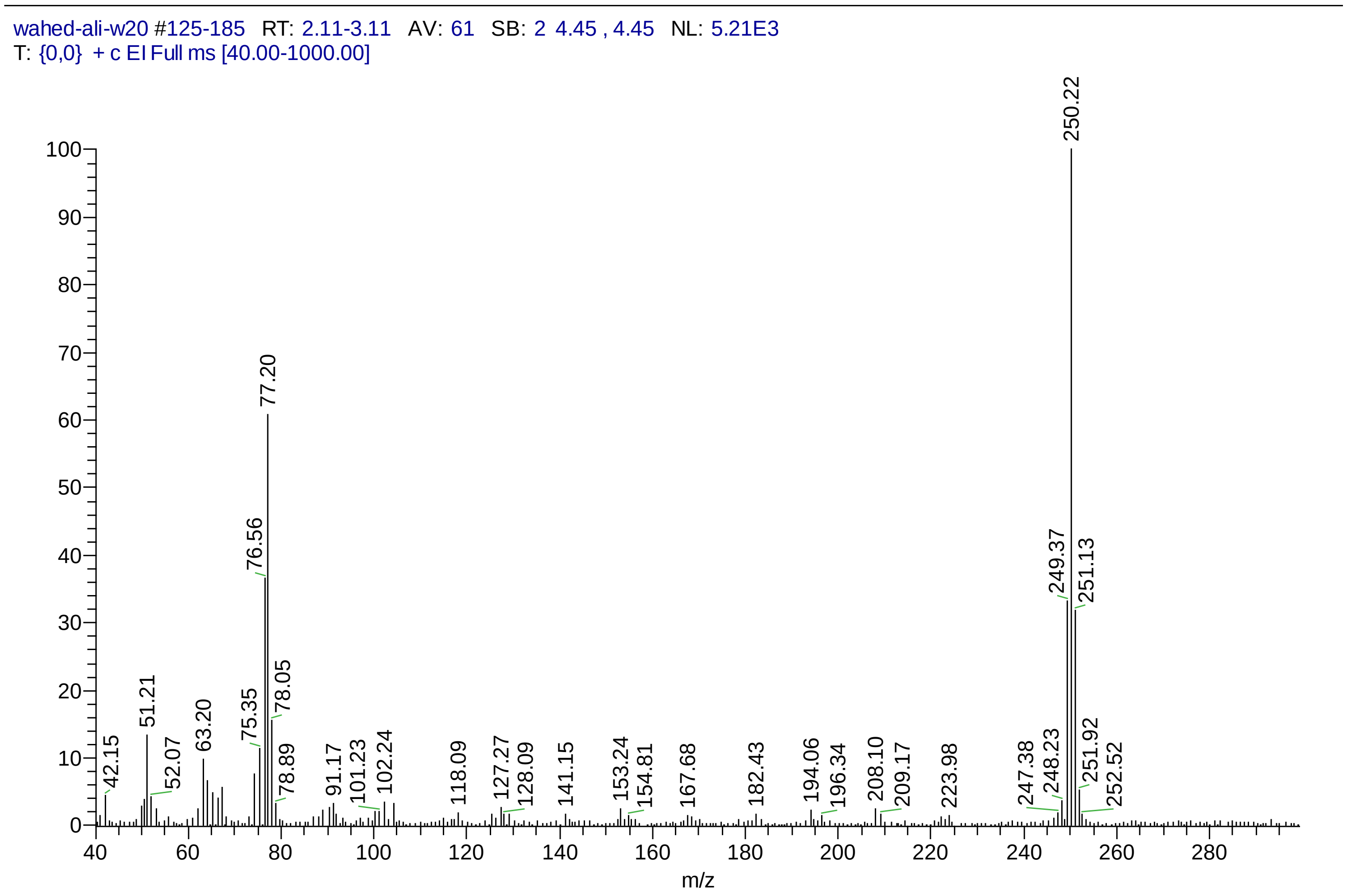

Mass of Compound 6a

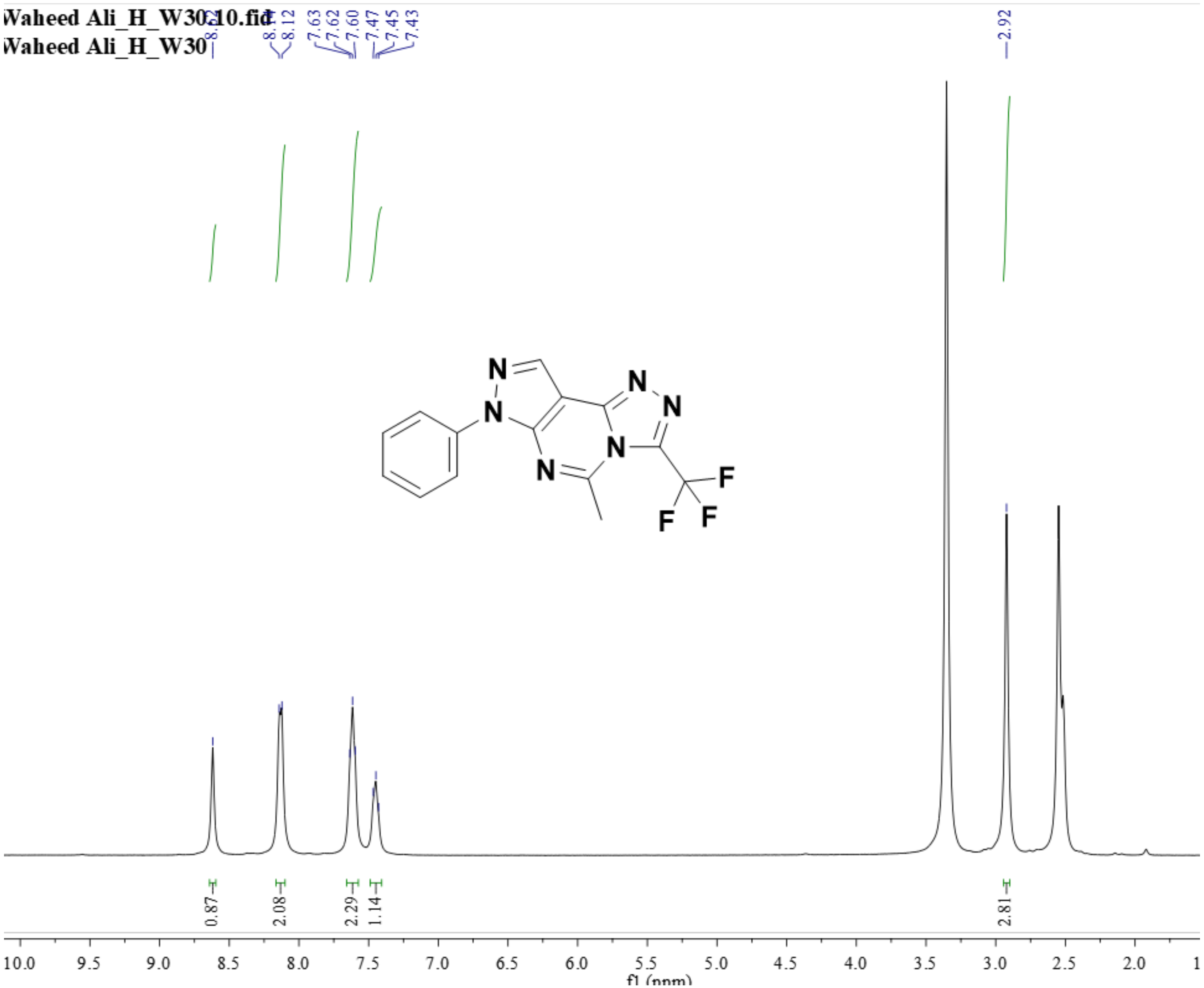

^{1}H NMR of compound 6b

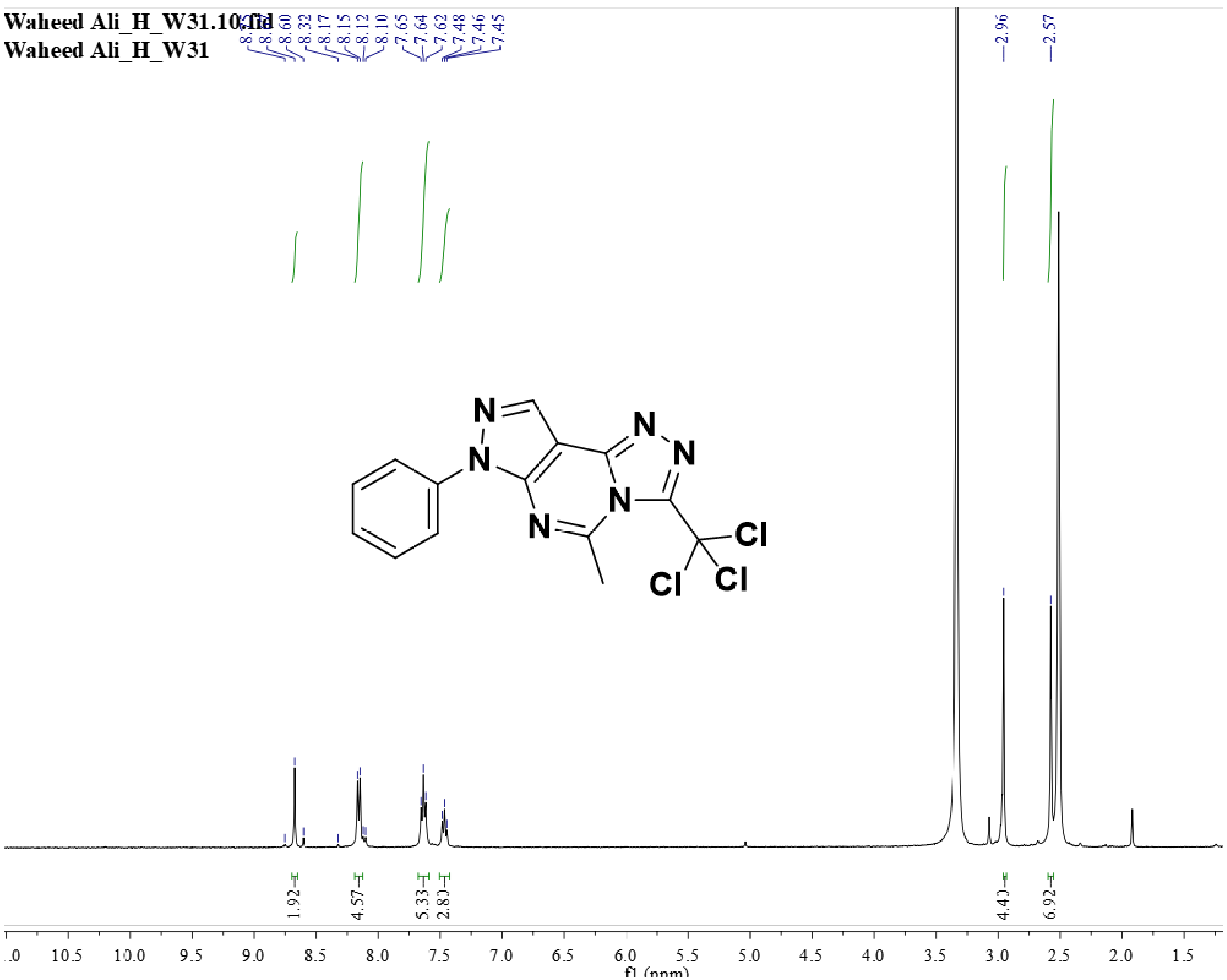

^{1}H NMR of compound 6c

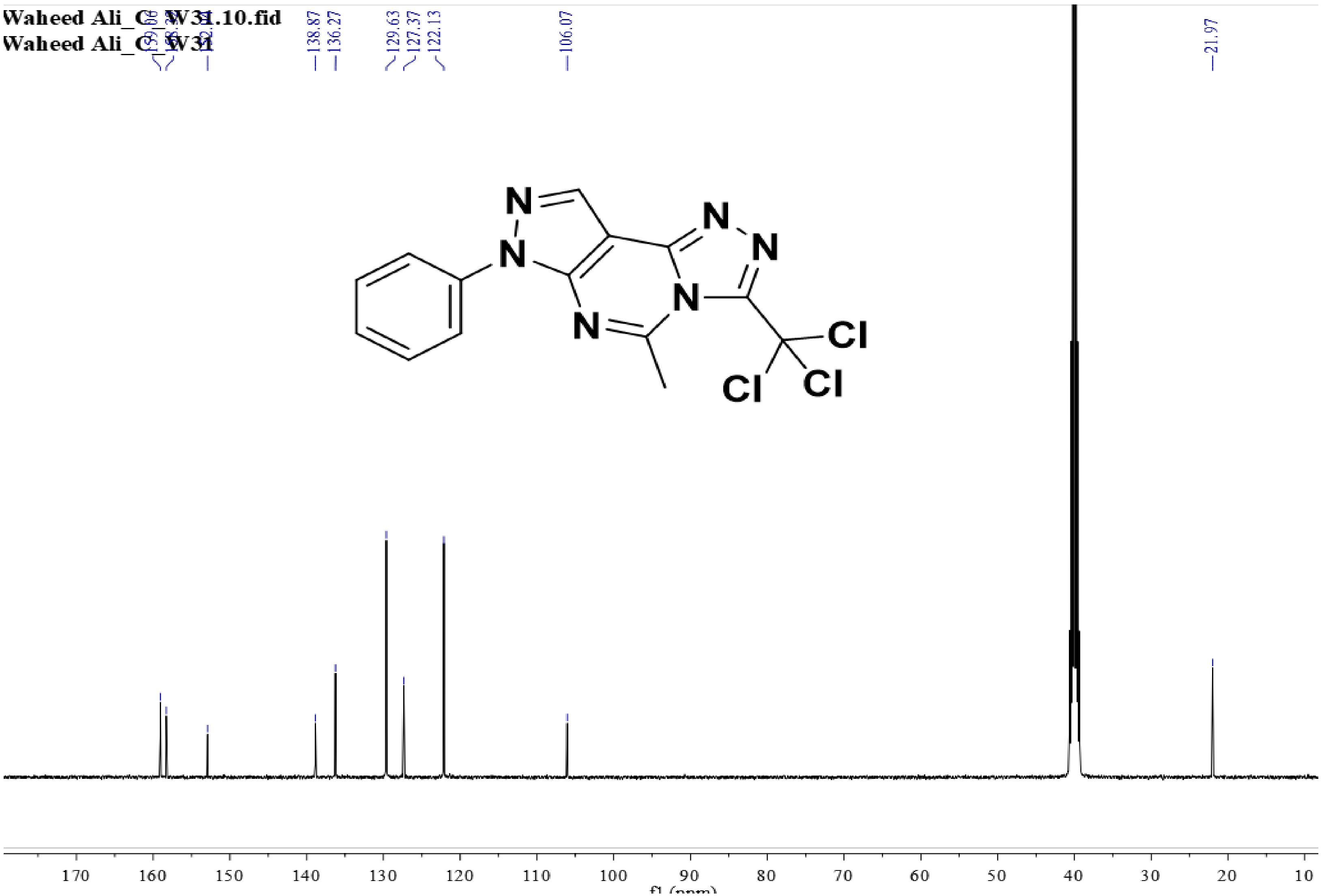

^{13}C NMR of compound 6c

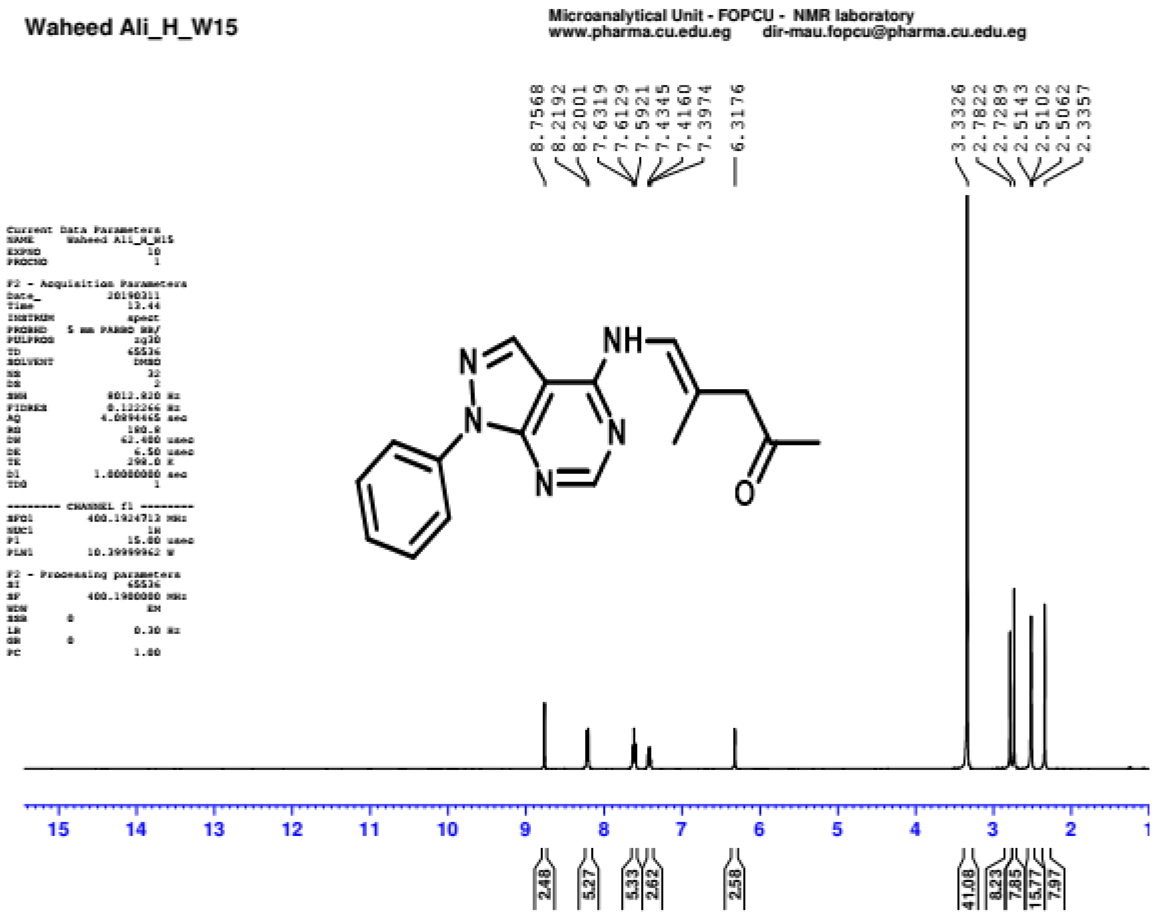

1H NMR of compound 6d

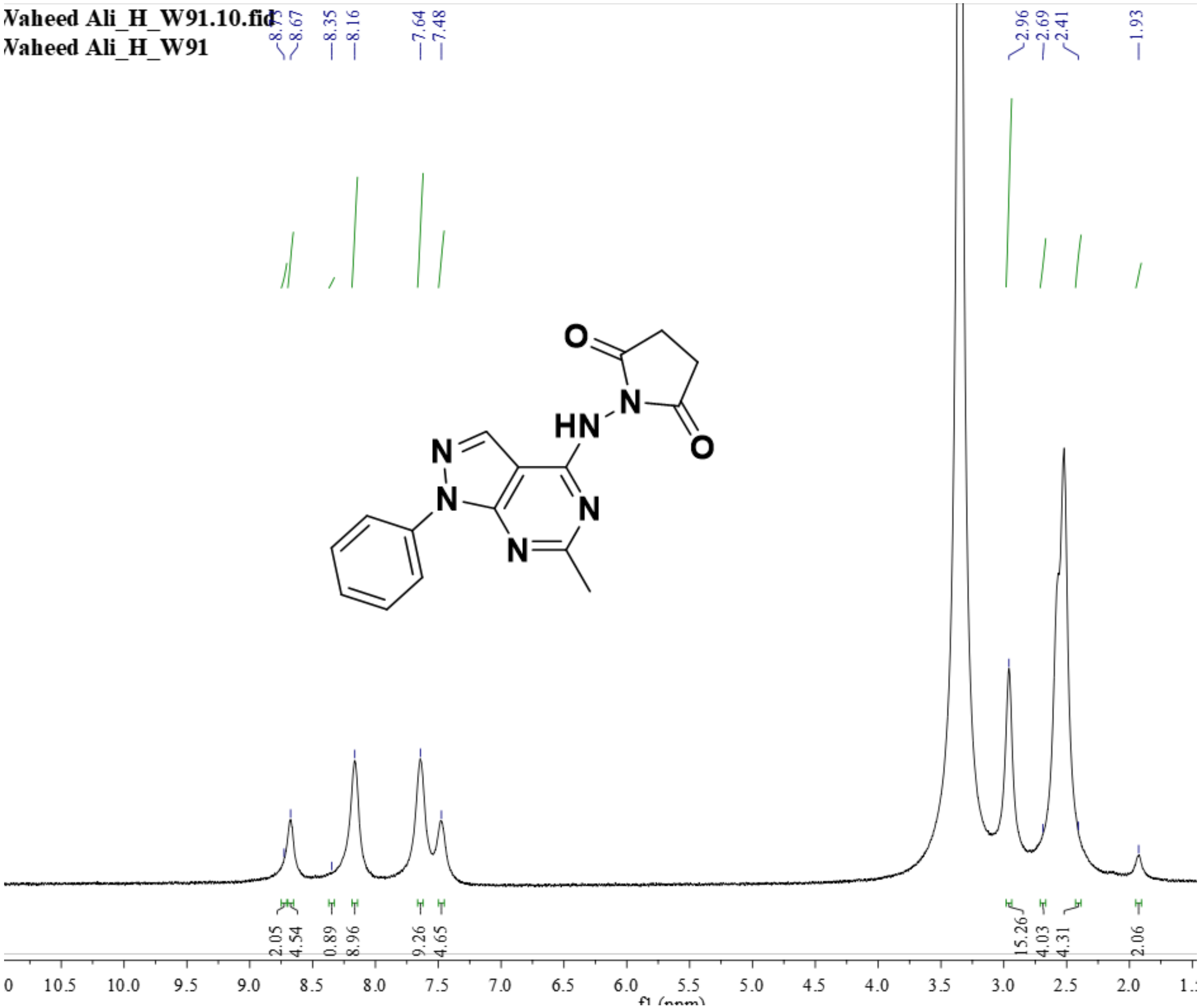

^{1}H NMR of compound 7

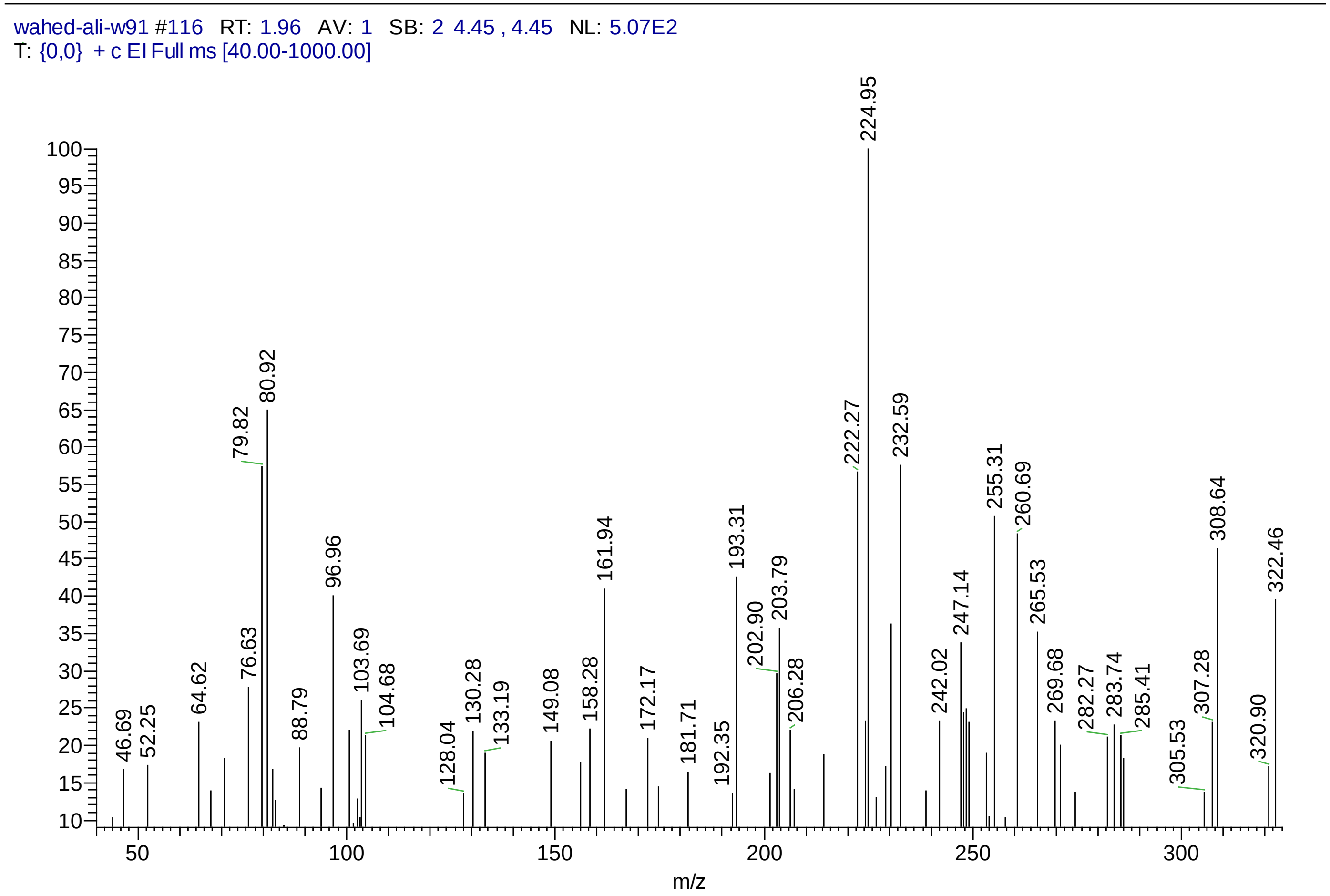

Mass of compound 7

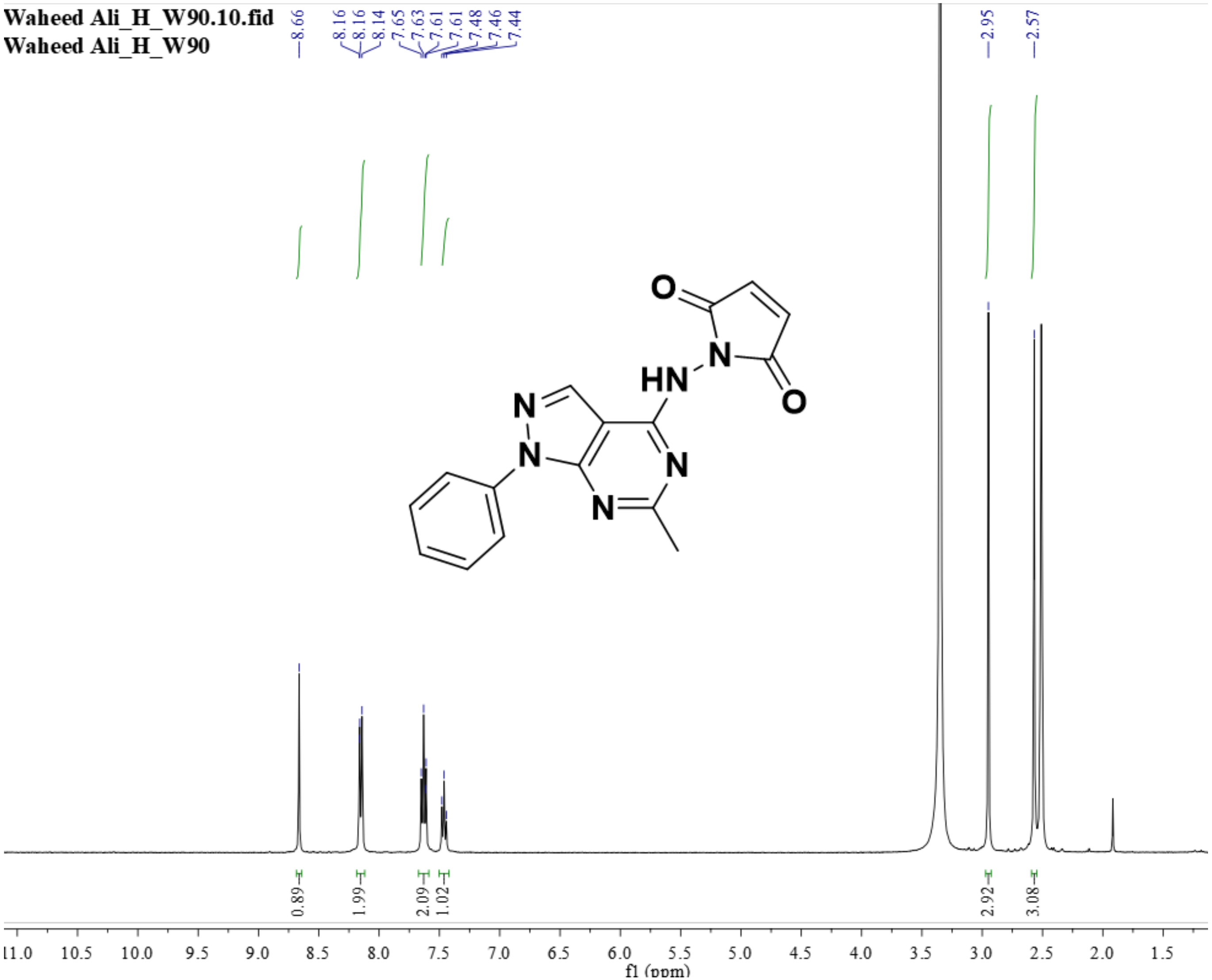

^{1}H NMR of compound 8

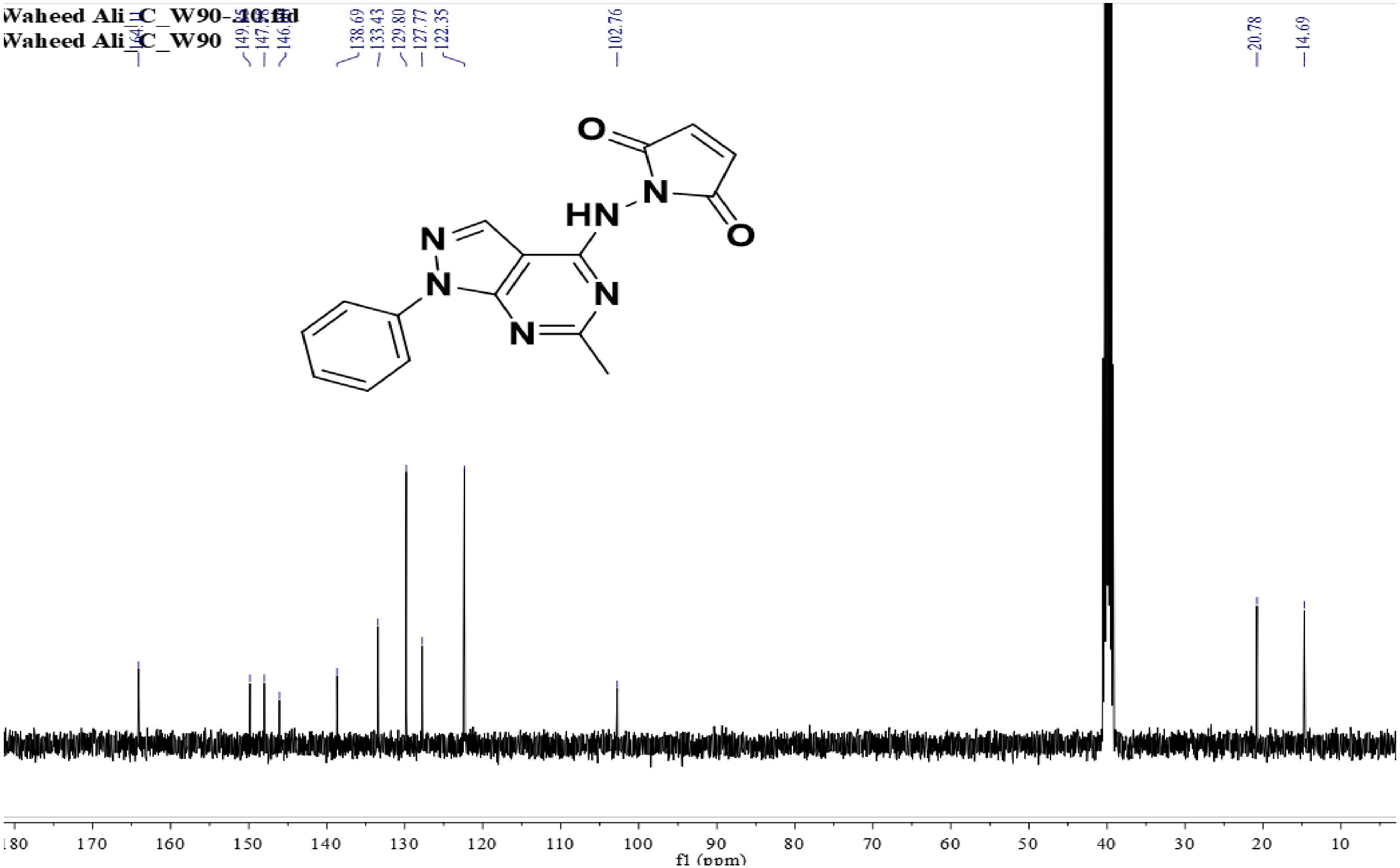

^{13}C NMR of compound 8

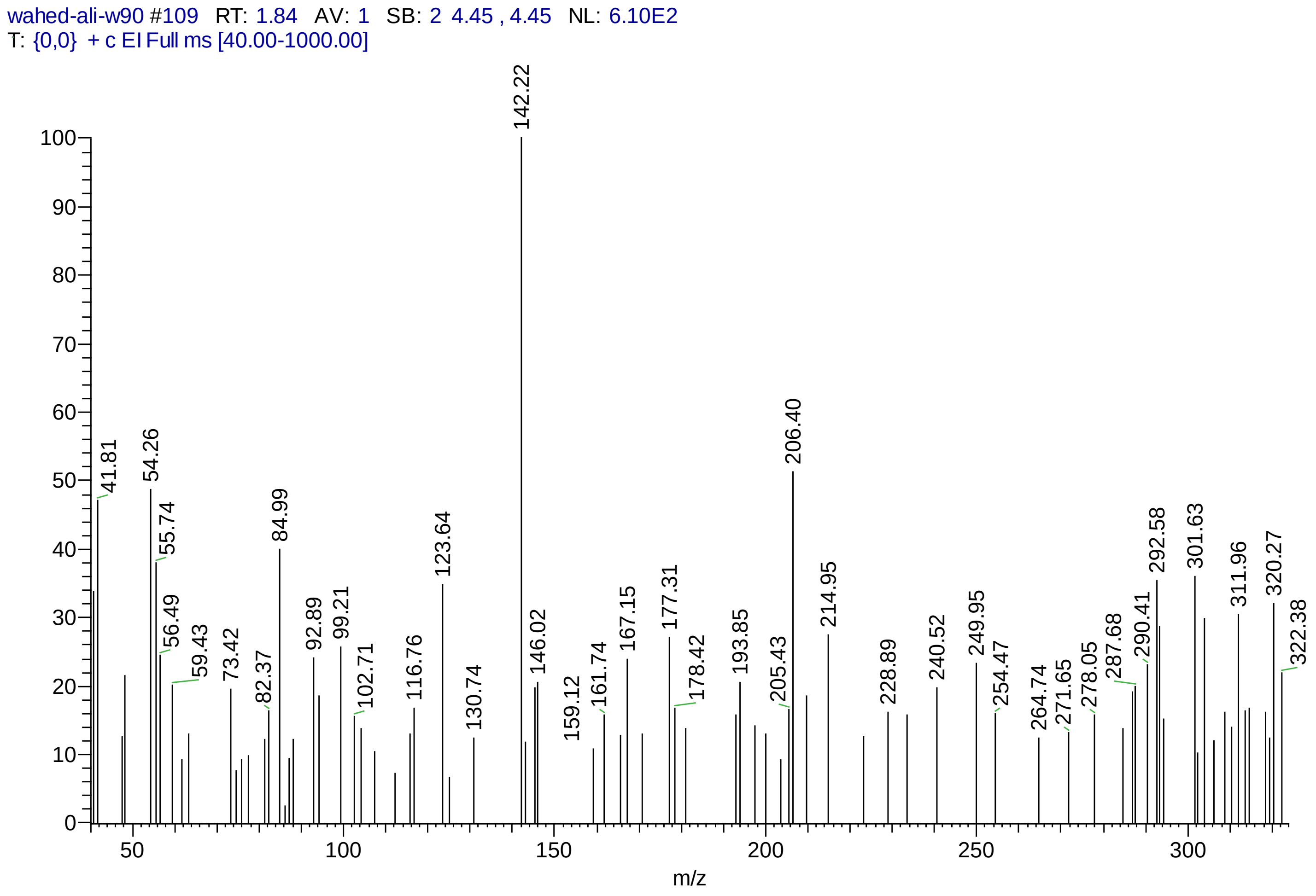

Mass of compound 8

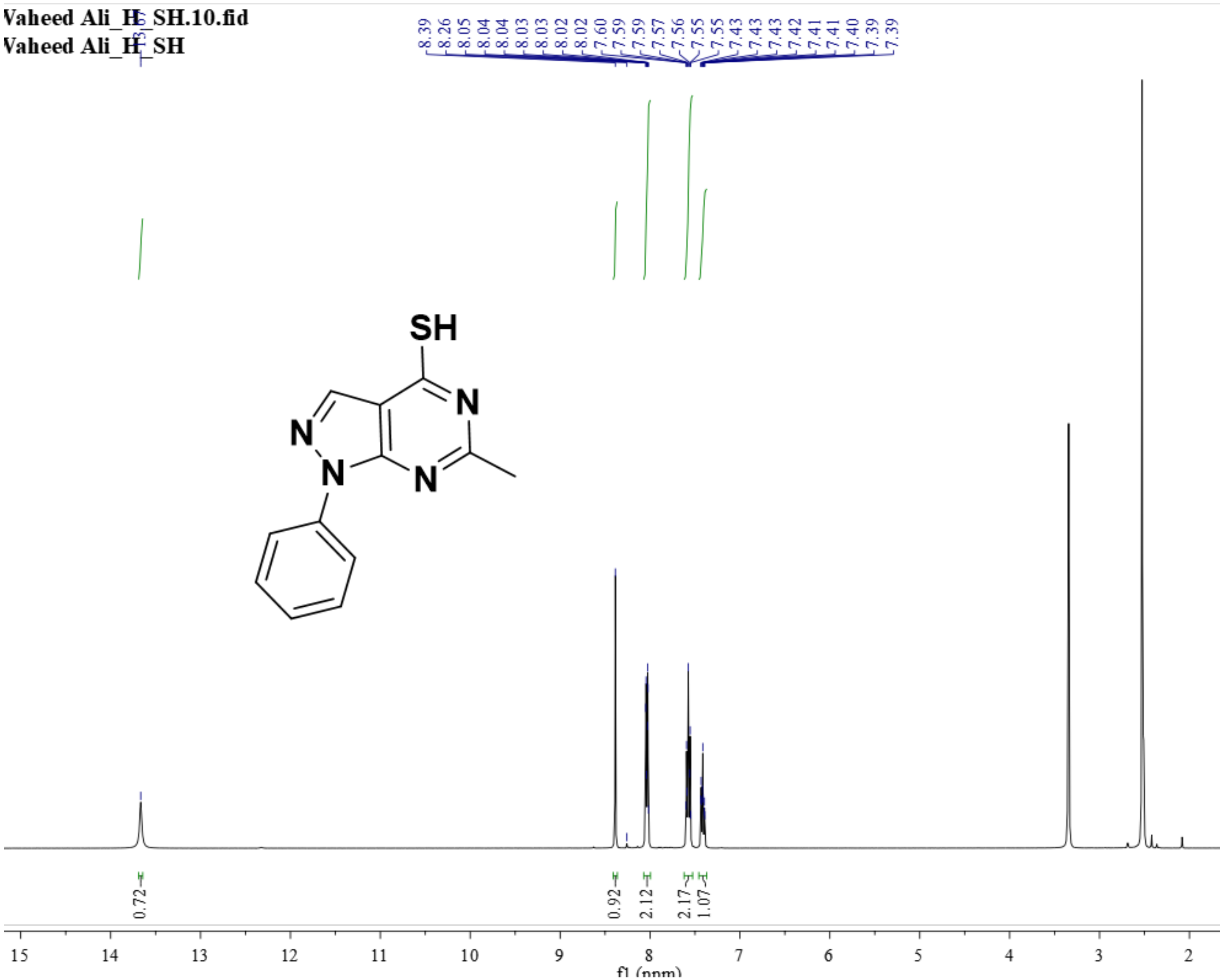

^{1}H NMR of compound 9

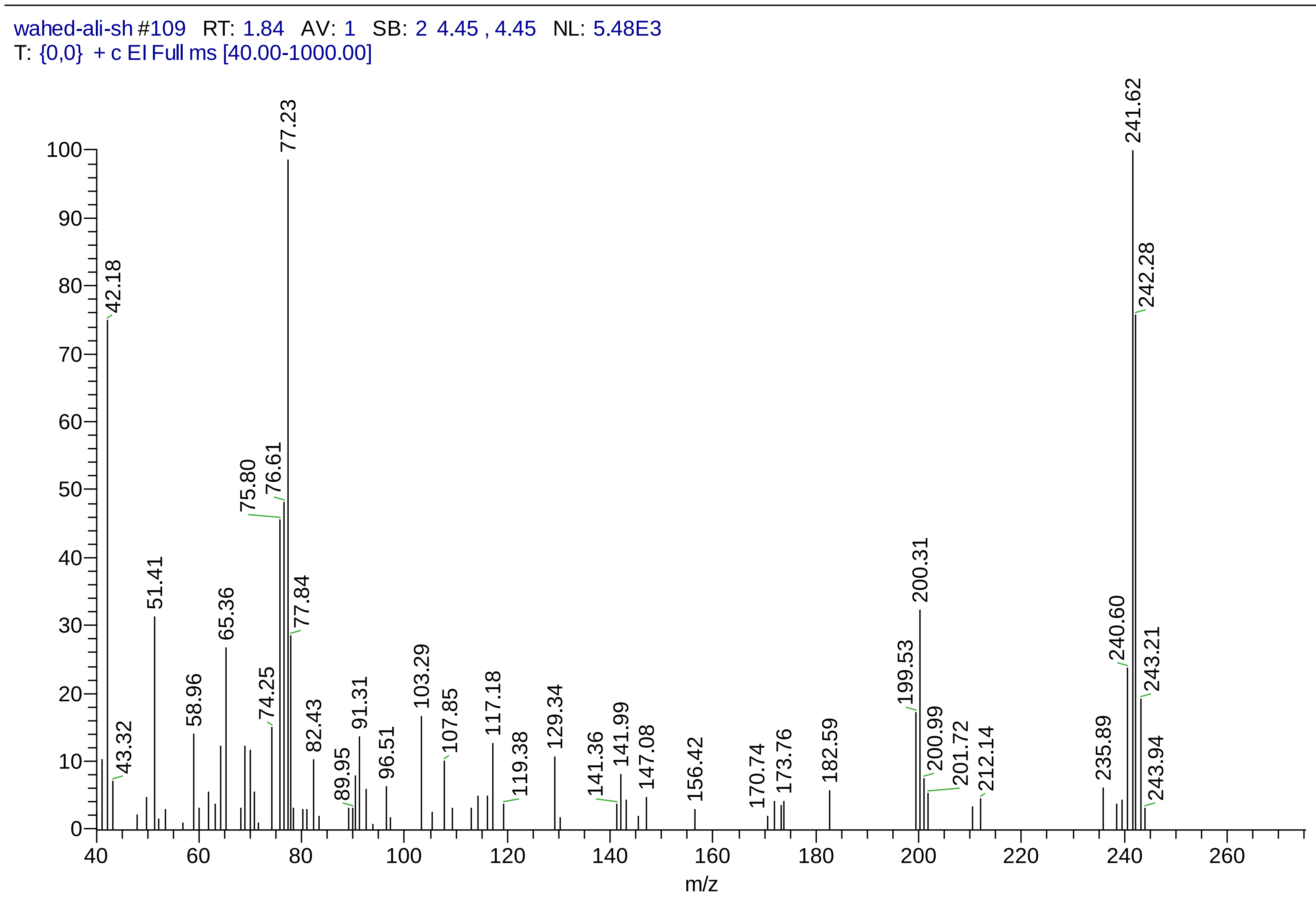

Mass of compound 9

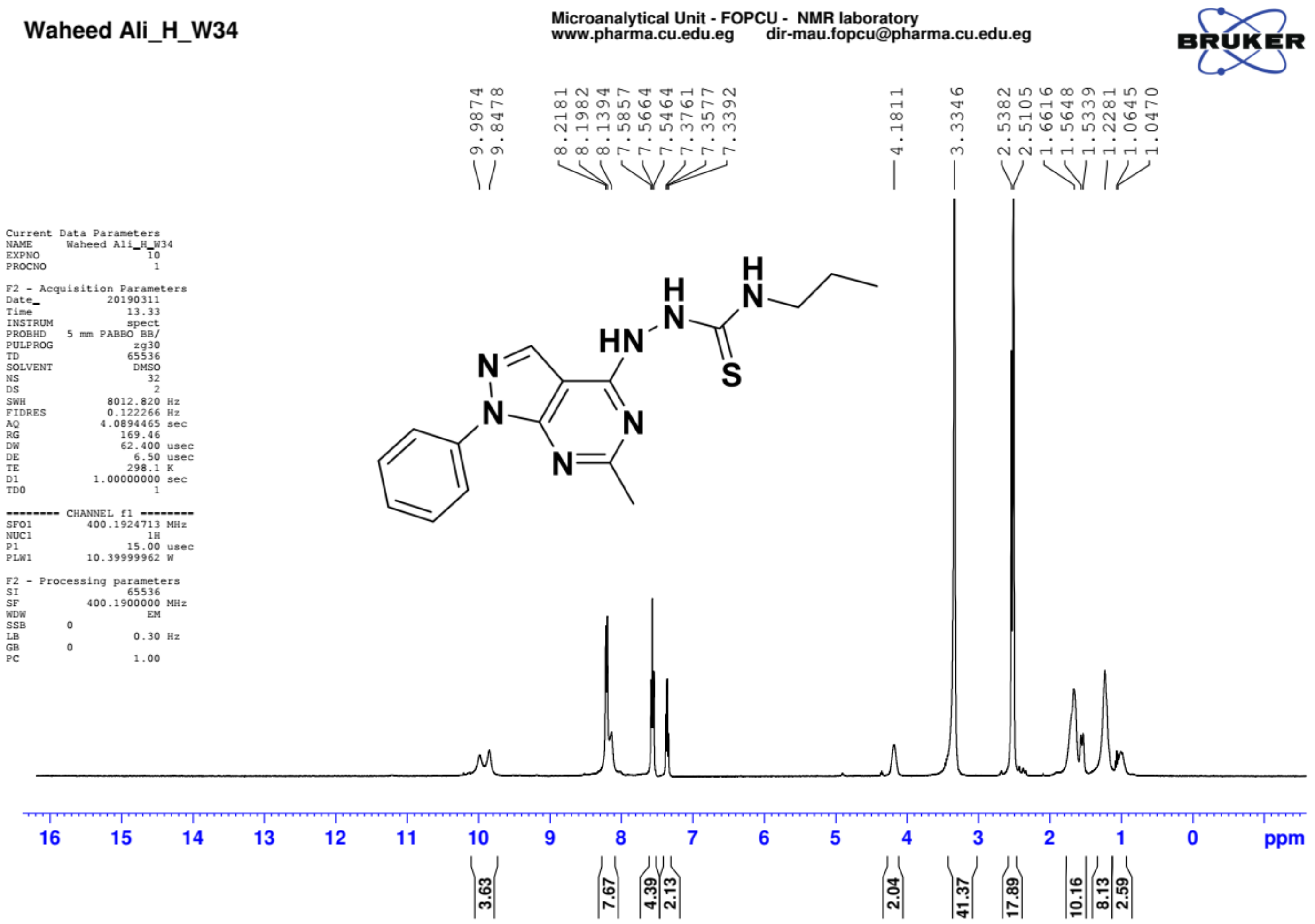

^{1}H NMR of compound 9b

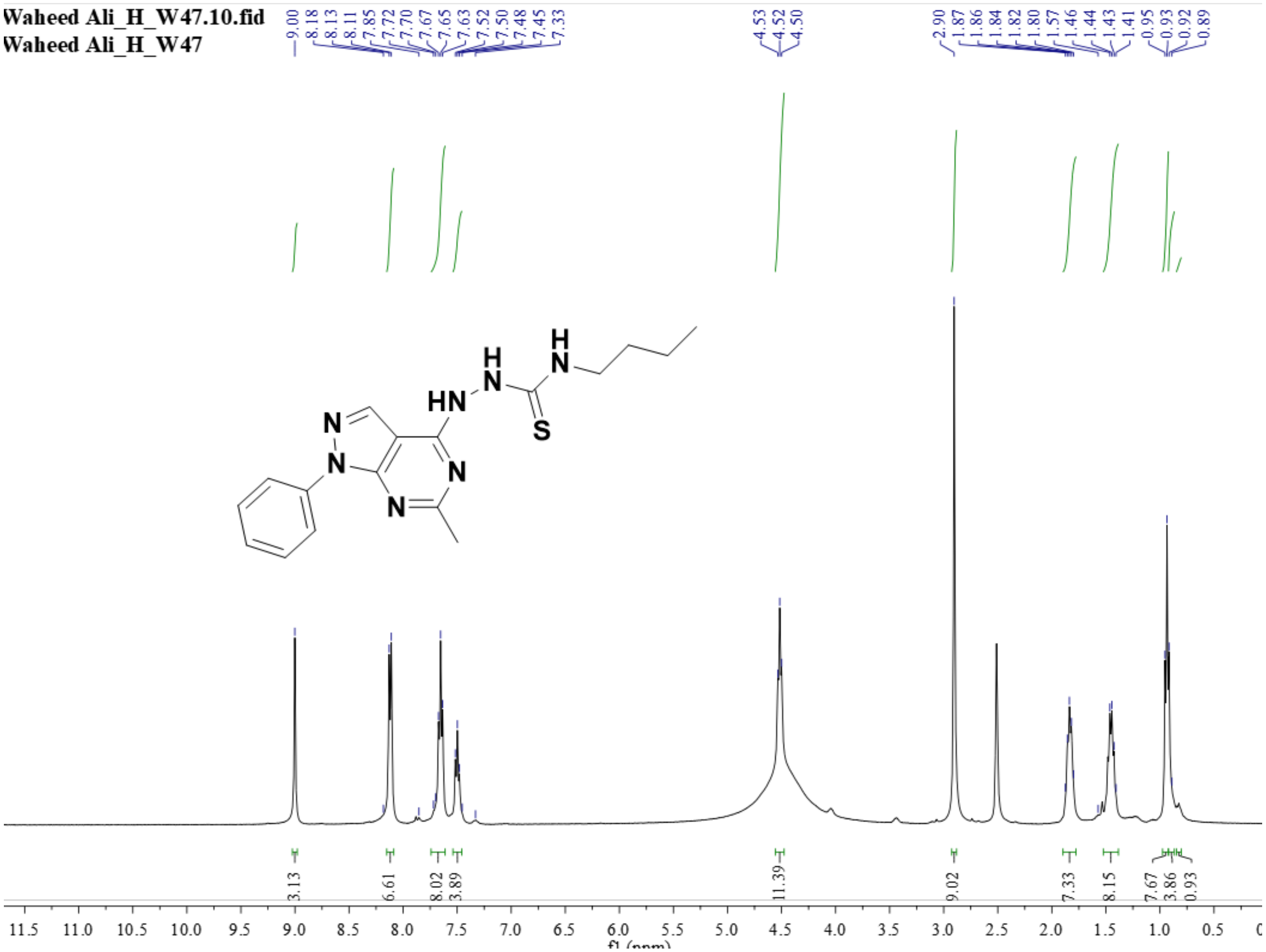

^{1}H NMR of compound 9c

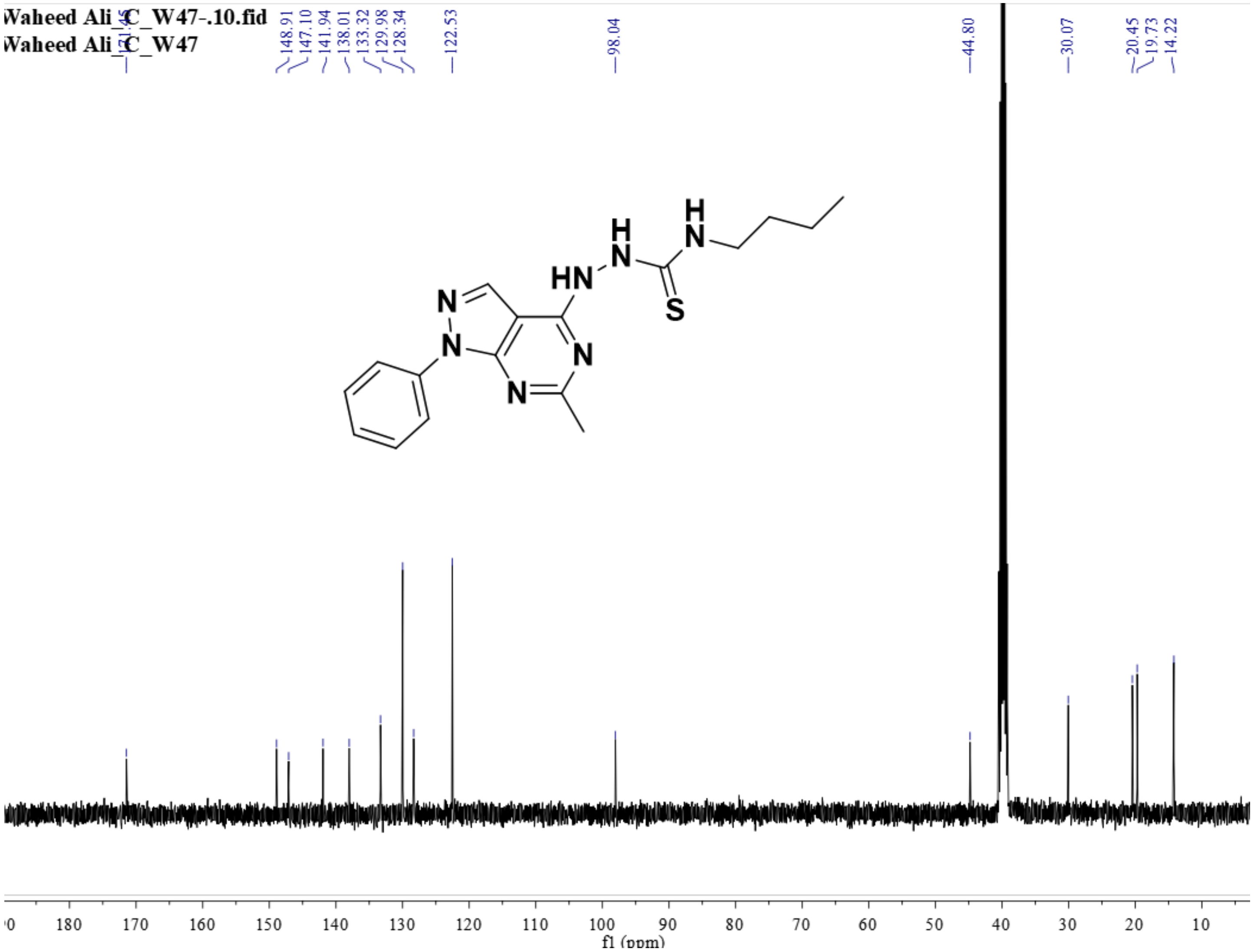

^{13}C NMR of compound 9c

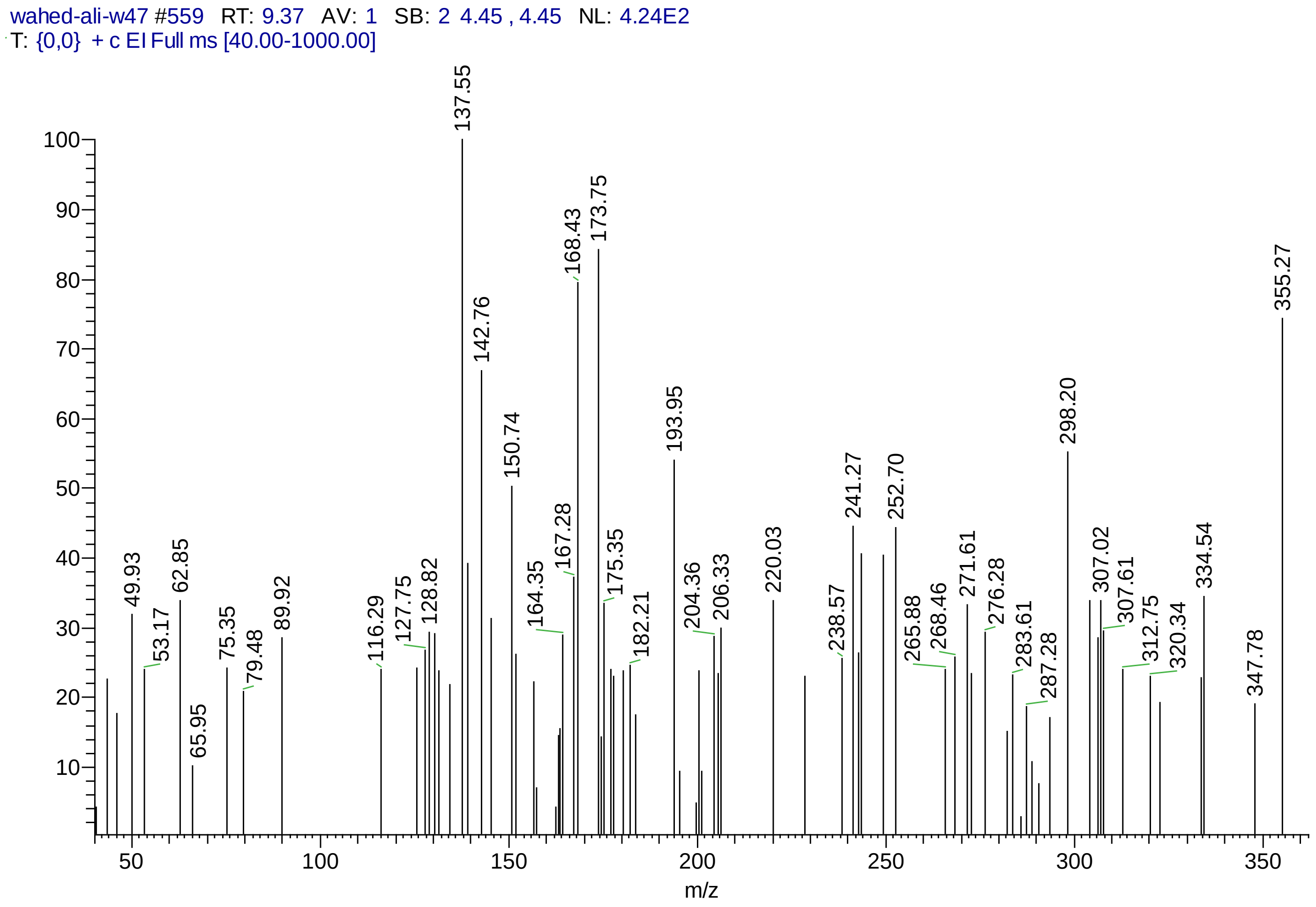

Mass of compound 9c

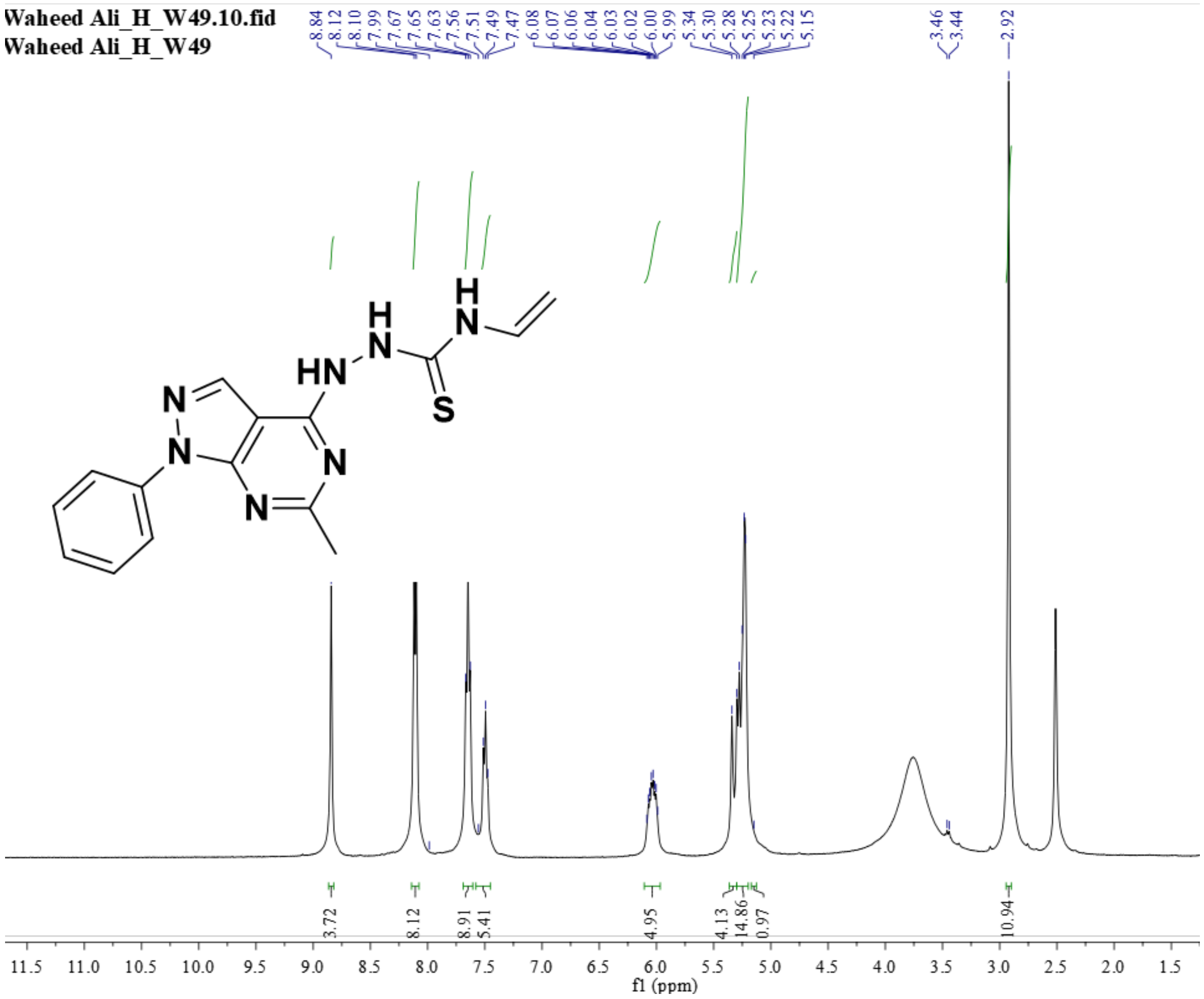

^{1}H NMR of compound 9d

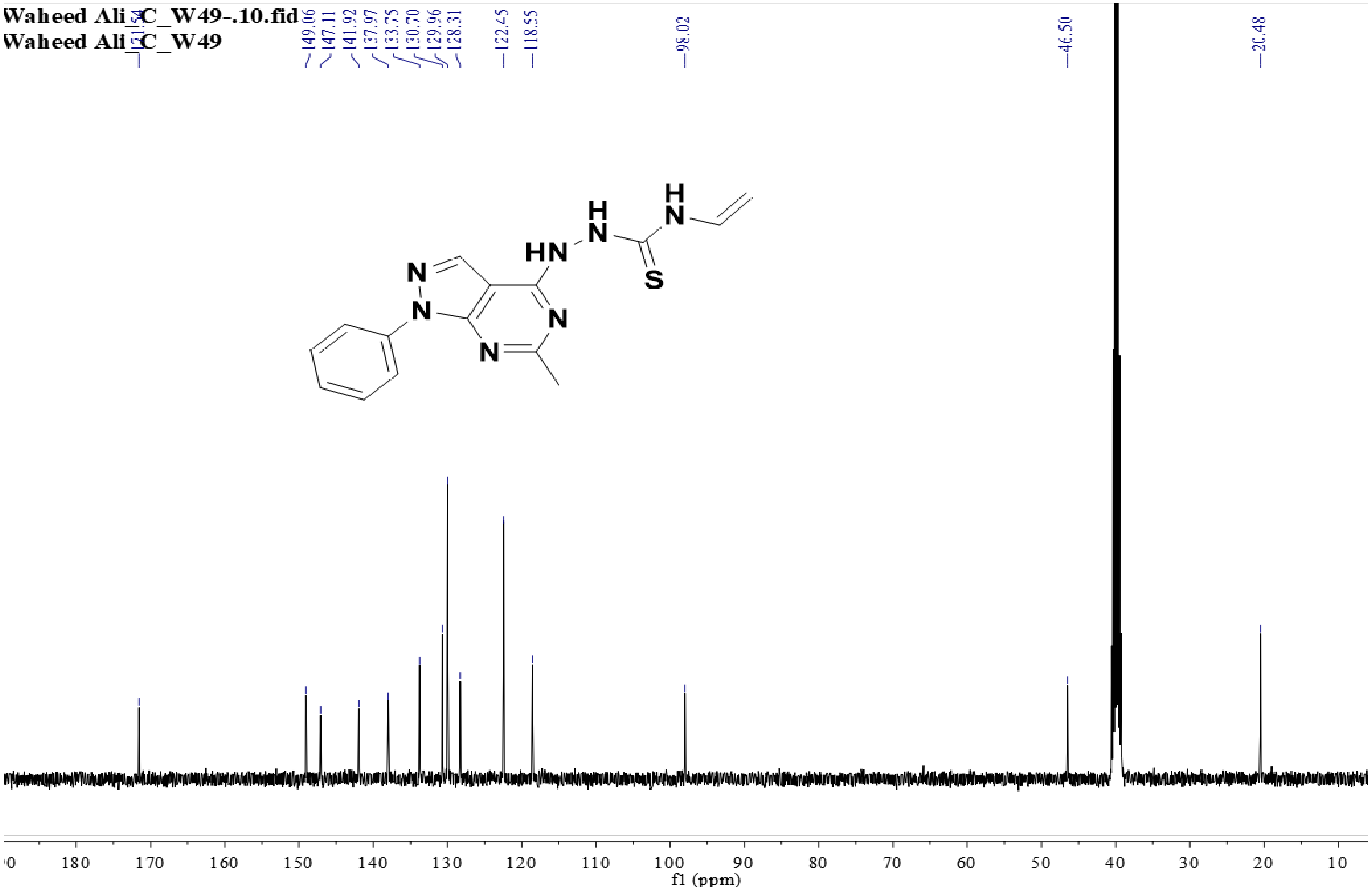

^{13}C NMR of compound 9d

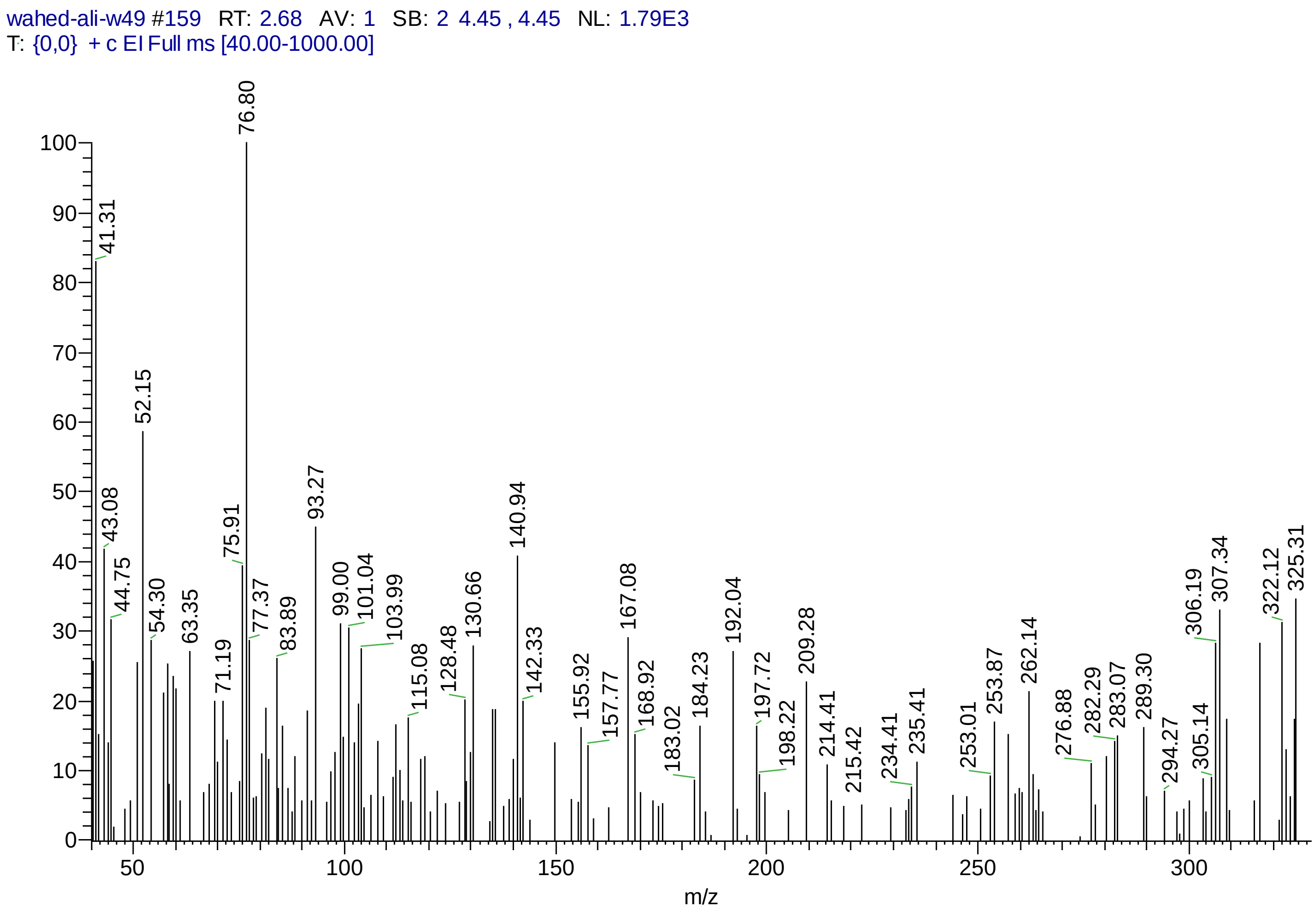

Mass of compound 9d

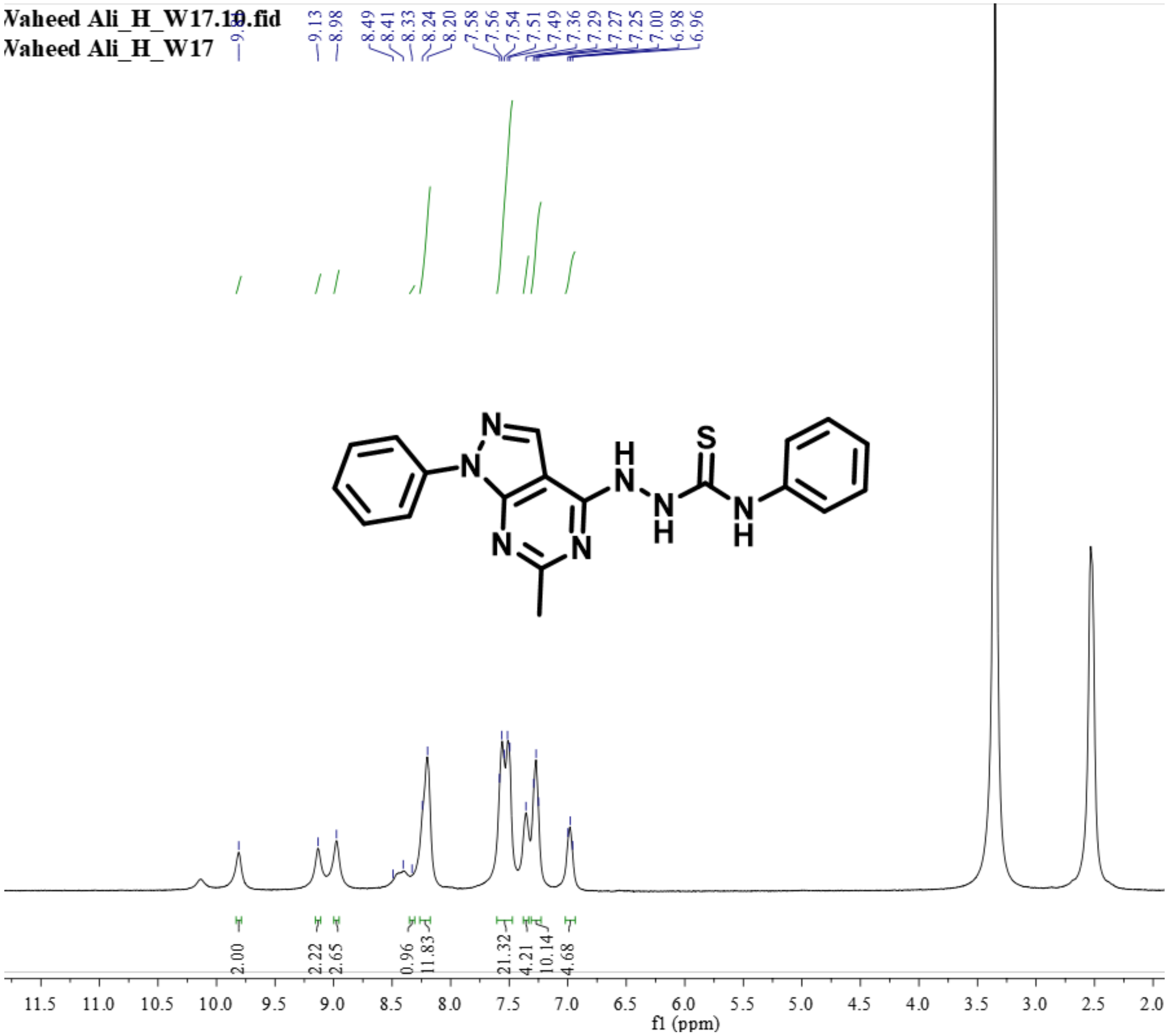

^{1}H NMR of compound 9e

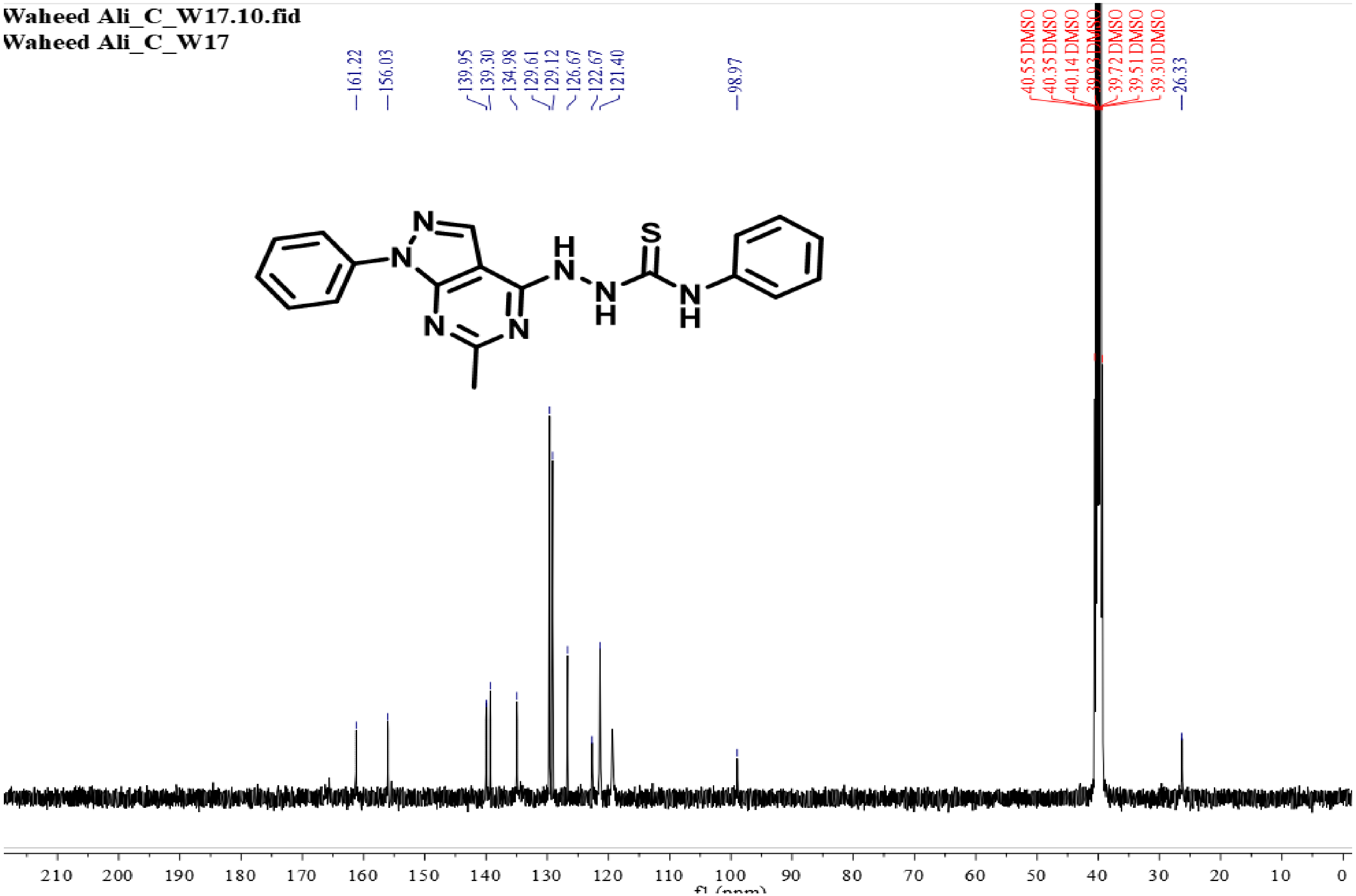

^{13}C of Compound 9e

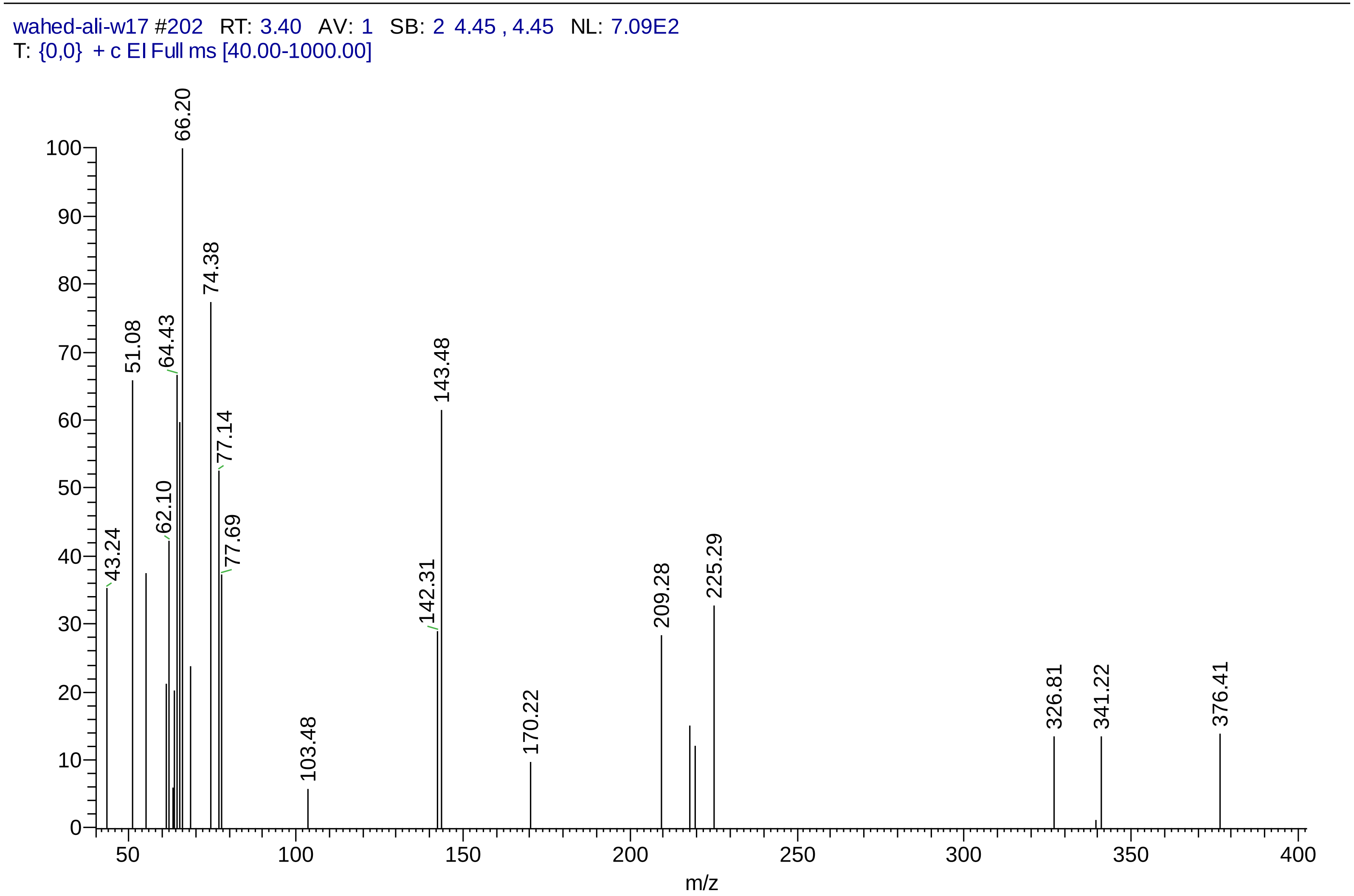

Mass of Compound 9e

SAMPLE INFORMATION

Sample Name:	W-17	Acquired By:	System
Sample Type:	Unknown	Sample Set Name:	errere
Vial:	7	Acq. Method Set:	FTANinstrument
Injection #:	1	Processing Method:	Default
Injection Volume:	10.00 ul	Channel Name:	210.0nm
Run Time:	26.0 Minutes	Proc. Chnl. Descr.:	996 PDA 210.0 nm (PDA 210.0 to 800.0 nm at
Date Acquired:	9/23/2023 7:42:44 PM EEST		
Date Processed:	9/24/2023 10:42:39 AM EEST		

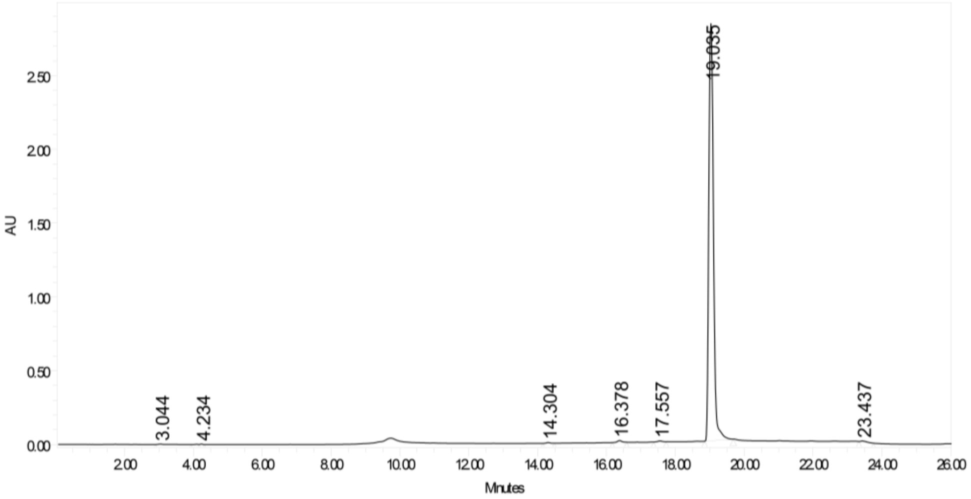

	RT	Area	%Area	Height
1	3.044	27972	0.10	2849
2	4.234	12902	0.05	3523
3	14.304	35493	0.13	4965
4	16.378	109738	0.41	12316
5	17.557	56735	0.21	6178
6	19.035	26364698	98.84	2830357
7	23.437	67141	0.25	6002

HPLC of Compound 9e

Researcher	: Dr.Hany Emary email: jan_25_misr@yahoo.com mob.+966534391522
Assay	: EGFR inh.assay
Samples	: 03 compounds
Cell lines	: ---
Ref.	: ---
Date	: 30-12-2021
Reader	: Tecan Spark Reader
Kit used	: ---.
Solvent	: DMSO

Lab Report

ser	Compound		EGFR	SD
	code	MW g/mol	IC50 ug/ml	±
1	5i		0.064	0.004
2	5b		0.126	0.008
3	9e		0.289	0.018
***	Erlotinib		0.052	0.003

Detailed Results

EGFR		[redacted]	[redacted]						[redacted]	[redacted]	[redacted]	[redacted]
		[redacted]		[redacted]	[redacted]		[redacted]		[redacted]		[redacted]	
code	IC50	conc	log	%inh	T2	T1	ΔT	RFU2	RFU1	ΔRFU	slope	K.Activity
5i		10	1	88	30	0	30	11.55	0	11.55	3.33333	13.86001
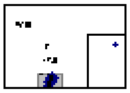												
		1	0	73	30	0	30	26.51	0	26.51	3.33333	31.81203
		0.1	-1	52	30	0	30	47.82	0	47.82	3.33333	57.38406
		0.01	-2	36	30	0	30	64.35	0	64.35	3.33333	77.22008
EC				0	30	0	30	100	0	100	3.33333	120
		[redacted]		[redacted]	[redacted]		[redacted]		[redacted]		[redacted]	

code	IC50	conc	log	%inh	T2	T1	ΔT	RFU2	RFU1	ΔRFU	slope	K.Activity
5b		10	1	87	30	0	30	13.05	0	13.05	3.33333	15.66002
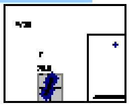												
		1	0	71	30	0	30	28.92	0	28.92	3.33333	34.70403
		0.1	-1	46	30	0	30	54.36	0	54.36	3.33333	65.23207
		0.01	-2	28	30	0	30	71.51	0	71.51	3.33333	85.81209
EC				0	30	0	30	100	0	100	3.33333	120
		[redacted]		[redacted]	[redacted]		[redacted]		[redacted]		[redacted]	

code	IC50	conc	log	%inh	T2	T1	ΔT	RFU2	RFU1	ΔRFU	slope	K.Activity
9e		10	1	83	30	0	30	16.81	0	16.81	3.33333	20.17202
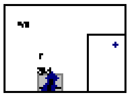												
		1	0	58	30	0	30	42.31	0	42.31	3.33333	50.77205
		0.1	-1	42	30	0	30	57.84	0	57.84	3.33333	69.40807
		0.01	-2	20	30	0	30	79.82	0	79.82	3.33333	95.7841
EC				0	30	0	30	100	0	100	3.33333	120
		[redacted]		[redacted]	[redacted]		[redacted]		[redacted]		[redacted]	

code	IC50	conc	log	%inh	T2	T1	ΔT	RFU2	RFU1	ΔRFU	slope	K.Activity
Erlotinib		10	1	92	30	0	30	8.35	0	8.35	3.33333	10.02001
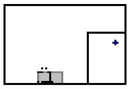												
		1	0	81	30	0	30	19.16	0	19.16	3.33333	22.99202
		0.1	-1	56	30	0	30	44.39	0	44.39	3.33333	53.26805
		0.01	-2	34	30	0	30	66.36	0	66.36	3.33333	79.63208
EC				0	30	0	30	100	0	100	3.33333	120

**Researcher**	: Dr.Hany Emary email: jan_25_misr@yahoo.com mob. +201032706127
**Assay**	: EGFR-T490M kinase assay
**Samples**	: 04 compounds () .
Cell lines	: ---
**Ref.**	: ----
**Date**	: 015-09-2022
**Reader**	: Tecan-spark reader
**Kit used**	: BPS bioscience EGFR assay kit.
**Solvent**	: DMSO

Lab Report

Ser	**Sample**		**EGFR IC50 uM**	SD
	**code**	**M.W g/mol**	**T790M**	
1	5b	344	1.93	0.08
2	5i	342	0.56	0.04
***	Erlotenib	393.436	0.035	0.001

Detailed results

[redacted]

code	IC50	conc	log	%inh	T2	T1	ΔT	RFU2	RFU1	ΔRFU	slope	K.Activity
5b		10	1	87	30	0	30	13.04	0	13.04	3.333	15.648
		1	0	77	30	0	30	22.51	0	22.51	3.333	27.012
		0.1	-1	54	30	0	30	46.37	0	46.37	3.333	55.64401
		0.01	-2	37	30	0	30	62.96	0	62.96	3.333	75.55201
EC				0	30	0	30	100	0	100	3.333	120

[redacted]

code	IC50	conc.ng/ml	log conc	%inh	T2	T1	ΔT	RFU2	RFU1	ΔRFU	slope	K.Activity
5i		10	1	89	30	0	30	11.07	0	11.07	3.333	13.284
		1	0	76	30	0	30	24.04	0	24.04	3.333	28.848
		0.1	-1	50	30	0	30	49.76	0	49.76	3.333	59.71201
		0.01	-2	37	30	0	30	63.28	0	63.28	3.333	75.93601
EC				0	30	0	30	100	0	100	3.333	120

[redacted]

code	IC50	conc.ng/ml	log conc	%inh	T2	T1	ΔT	RFU2	RFU1	ΔRFU	slope	K.Activity
Erlotenib		10	1	94	30	0	30	6.49	0	6.49	3.333	7.788001
		1	0	83	30	0	30	16.81	0	16.81	3.333	20.172
		0.1	-1	61	30	0	30	38.76	0	38.76	3.333	46.512
		0.01	-2	48	30	0	30	52.41	0	52.41	3.333	62.89201
EC				0	30	0	30	100	0	100	3.333	120

[redacted]

Researcher	: Dr.Waheed Ali	email: dr.waheed202121@gmail.com mob.
Assay	: **TOPO IIB**	dr_elseba3y80@outlook.com
Samples	: 06 compounds	
Ref.	: ---	
Date	: 10-04-2021	
Reader	: ---	
Cell line	: ---	
Kit used	: Human DNA Topoisomerase 2B Kit	
Solvent	: DMSO	

Lab.Report

ser	Compound			TOP2a IC50	SD
	code	MW	conc ug	ug/ml	±
1	W3			**6.21**	0.37
2	5b			**37.3**	2.2
3	5i			**8.46**	0.5
4	W10			**5.38**	0.32
5	W11			**89.5**	5.26
6	9e			**9.24**	0.54
7	W35			**60.5**	3.56
***	Etoposide			**19.8**	1.16

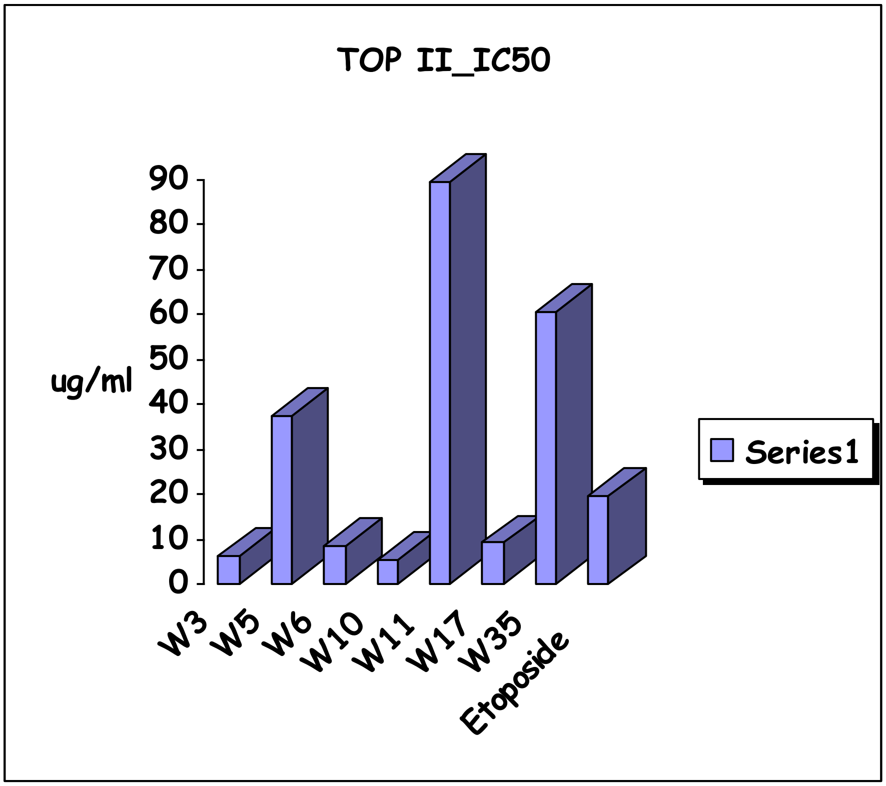

Detailed results

TOPO IiB

sample

code	IC50	conc	log	%inh
W3		100	2	71
		10	1	57
		1	0	34
		0.1	-1	16
EC				0

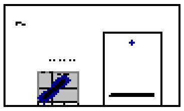

code	IC50	conc	log	%inh
5b		100	2	66
		10	1	31
		1	0	12
		0.1	-1	6.7
EC				0

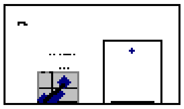

code	IC50	conc	log	%inh
5i		100	2	74
		10	1	51
		1	0	27
		0.1	-1	13
EC				0

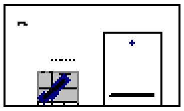

code	IC50	conc	log	%inh
W10		100	2	77
		10	1	62
		1	0	29
		0.1	-1	11
EC				0

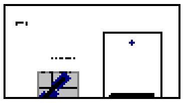

code	IC50	conc	log	%inh
W11		100	2	59
		10	1	24
		1	0	8.4
		0.1	-1	4.5
EC				0

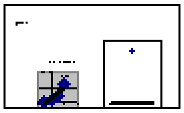

code	IC50	conc	log	%inh

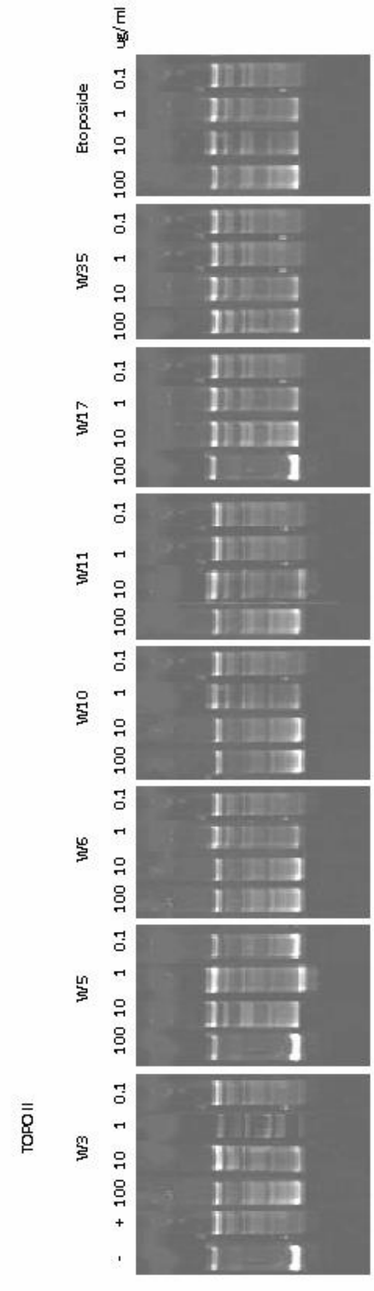

code	IC50	conc	log	%inh
9e		100	2	65
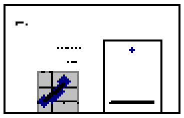		10	1	34
		1	0	16
		0.1	-1	4
EC				0
	[redacted]			

code	IC50	conc	log	%inh
W35		100	2	60
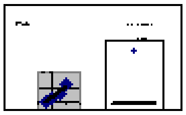		10	1	28
		1	0	15
		0.1	-1	3.7
EC				0
	[redacted]			

code	IC50	conc	log	%inh
Etoposide		100	2	69
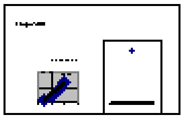		50	1	39
		25	0	20
		12.5	-1	7.5
EC				0
			[redacted]	

**W3**	y = 18.811x + 35.077
**W5**	y = 19.773x + 18.914
**W6**	y = 20.71x + 30.794
**W10**	y = 22.97x + 33.219
**W11**	y = 17.906x + 15.05
**W17**	y = 20.21x + 19.598
**W35**	y = 18.267x + 17.457
**Etoposide**	y = 20.241x + 23.76

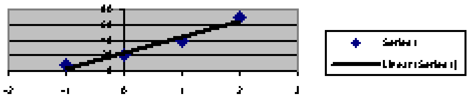

Researcher	: Dr.Waheed Ali	email: dr.waheed202121@gmail.com	mob.
Assay	: VEGFR2 enzyme assay		
Samples	: 07 compounds.		
Cell line	: ---		
Reference	: ---		
Date	: 10/04/2021		
Kit used	: ---		
Reader	: Tecan –spark reader BIOLINE ELISA READER wl 450 nm		
Solvent	: DMSO		
Assay samples	: ---		

Lab Report

ser	Compound		Results	SD
	code	MW g/mol	VEGFR2 IC50 ug/ml	±
1	W3		0.26	0.02
2	5b		5.42	0.32
3	5i		2.596	0.15
4	W10		0.119	0.01
5	W11		0.459	0.03
6	9e		6.094	0.36
7	W35		0.418	0.02
***	Sorafenib		0.034	0.002

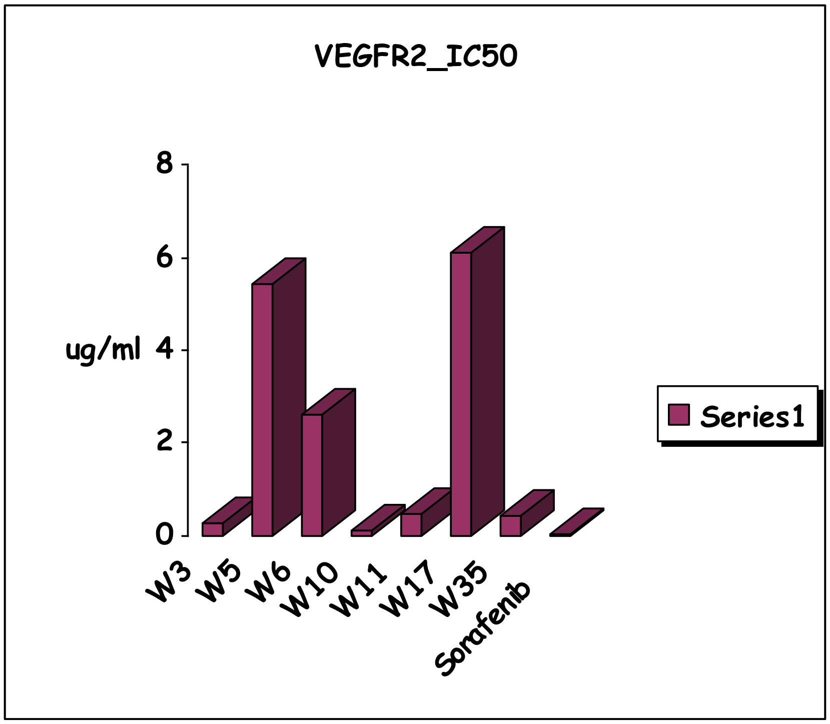

Detailed results

VEGFR2 [redacted]

sample

code	IC50	conc	log	%inh	T2	T1	ΔT	RFU2	RFU1	ΔRFU	slope	K.Activity	EC
W3		10	2	89	30	0	30	11.05	0	11.05	3.333	13.2613	120
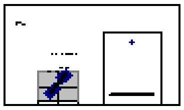		1	1	78	30	0	30	22.41	0	22.41	3.333	26.8947	120
		0.1	-1	43	30	0	30	57.36	0	57.36	3.333	68.8389	120
		0.01	-2	28	30	0	30	72.28	0	72.28	3.333	86.7447	120
EC				0	30	0	30	100	0	100	3.333	120	120

[redacted]

code	IC50	conc	log	%inh	T2	T1	ΔT	RFU2	RFU1	ΔRFU	slope	K.Activity	EC
5b		10	2	70	30	0	30	29.55	0	29.55	3.333	35.4635	120
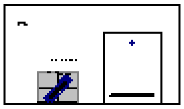		1	1	50	30	0	30	49.76	0	49.76	3.333	59.718	120
		0.1	-1	25	30	0	30	75.02	0	75.02	3.333	90.033	120
		0.01	-2	13	30	0	30	86.51	0	86.51	3.333	103.822	120
EC				0	30	0	30	100	0	100	3.333	120	120

[redacted]

code	IC50	conc	log	%inh	T2	T1	ΔT	RFU2	RFU1	ΔRFU	slope	K.Activity	EC
5i		10	2	84	30	0	30	15.71	0	15.71	3.333	18.8539	120
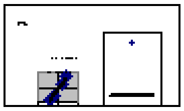		1	1	58	30	0	30	42.31	0	42.31	3.333	50.7771	120
		0.1	-1	19	30	0	30	81.38	0	81.38	3.333	97.6658	120
		0.01	-2	7.5	30	0	30	92.51	0	92.51	3.333	111.023	120
EC				0	30	0	30	100	0	100	3.333	120	120

[redacted]

code	IC50	conc	log	%inh	T2	T1	ΔT	RFU2	RFU1	ΔRFU	slope	K.Activity	EC
W10		10	2	91	30	0	30	9.36	0	9.36	3.333	11.2331	120
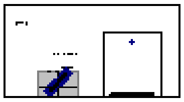		1	1	76	30	0	30	24.39	0	24.39	3.333	29.2709	120
		0.1	-1	55	30	0	30	45.12	0	45.12	3.333	54.1494	120
		0.01	-2	31	30	0	30	69.24	0	69.24	3.333	83.0963	120
EC				0	30	0	30	100	0	100	3.333	120	120

[redacted]

code	IC50	conc	log	%inh	T2	T1	ΔT	RFU2	RFU1	ΔRFU	slope	K.Activity	EC
W11		10	2	87	30	0	30	13.06	0	13.06	3.333	15.6736	120
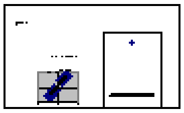		1	1	71	30	0	30	29.45	0	29.45	3.333	35.3435	120
		0.1	-1	41	30	0	30	59.32	0	59.32	3.333	71.1911	120
		0.01	-2	23	30	0	30	76.84	0	76.84	3.333	92.2172	120
EC				0	30	0	30	100	0	100	3.333	120	120

[redacted]

code	IC50	conc	log	%inh	T2	T1	ΔT	RFU2	RFU1	ΔRFU	slope	K.Activity	EC
9e		10	2	72	30	0	30	28.23	0	28.23	3.333	33.8794	120
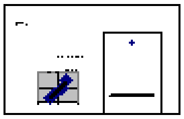		1	1	46	30	0	30	54.19	0	54.19	3.333	65.0345	120

		0.1	-1	26	30	0	30	73.82	0	73.82	3.333	88.5929	120
		0.01	-2	14	30	0	30	86.47	0	86.47	3.333	103.774	120
EC				0	30	0	30	100	0	100	3.333	120	120

code	IC50	conc	log	%inh	T2	T1	ΔT	RFU2	RFU1	ΔRFU	slope	K.Activity	EC
W35		10	2	87	30	0	30	12.74	0	12.74	3.333	15.2895	120
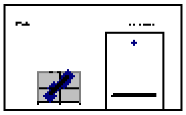													
		1	1	68	30	0	30	31.52	0	31.52	3.333	37.8278	120
		0.1	-1	45	30	0	30	55.29	0	55.29	3.333	66.3546	120
		0.01	-2	23	30	0	30	77.29	0	77.29	3.333	92.7573	120
EC				0	30	0	30	100	0	100	3.333	120	120

code	IC50	conc	log	%inh	T2	T1	ΔT	RFU2	RFU1	ΔRFU	slope	K.Activity	EC
Sorafenib		10	2	91	30	0	30	8.51	0	8.51	3.333	10.213	120
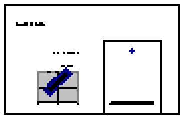													
		1	1	82	30	0	30	17.81	0	17.81	3.333	21.3741	120
		0.1	-1	63	30	0	30	36.84	0	36.84	3.333	44.2124	120
		0.01	-2	38	30	0	30	62.41	0	62.41	3.333	74.8995	120
EC				0	30	0	30	100	0	100	3.333	120	120

**W3**	y = 15.743x + 59.221
**W5**	y = 13.919x + 39.784
**W6**	y = 19.269x + 42.017
**W10**	y = 14.05x + 62.969
**W11**	y = 15.745x + 55.328
**W17**	y = 13.612x + 39.316
**W35**	y = 15.289x + 55.786
**Sorafenib**	y = 12.684x + 68.604

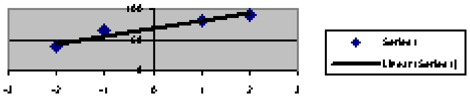

Supplement: Supplementary file 1 [file ijms-24-15026-s001.zip › ijms-2647906-supplementary.pdf]
